# Understanding psychiatric disorder by capturing ecologically relevant features of learning and decision-making

**DOI:** 10.1016/j.bbr.2017.09.050

**Published:** 2018-12-14

**Authors:** Jacqueline Scholl, Miriam Klein-Flügge

**Affiliations:** Department of Experimental Psychology, University of Oxford, Tinsley Building, Mansfield Road, Oxford, OX1 3SR, United Kingdom

**Keywords:** Reinforcement learning, Decision-making, Computational psychiatry

## Abstract

Recent research in cognitive neuroscience has begun to uncover the processes underlying increasingly complex voluntary behaviours, including learning and decision-making. Partly this success has been possible by progressing from simple experimental tasks to paradigms that incorporate more ecological features. More specifically, the premise is that to understand cognitions and brain functions relevant for real life, we need to introduce some of the ecological challenges that we have evolved to solve. This often entails an increase in task complexity, which can be managed by using computational models to help parse complex behaviours into specific component mechanisms. Here we propose that using computational models with tasks that capture ecologically relevant learning and decision-making processes may provide a critical advantage for capturing the mechanisms underlying symptoms of disorders in psychiatry. As a result, it may help develop mechanistic approaches towards diagnosis and treatment. We begin this review by mapping out the basic concepts and models of learning and decision-making. We then move on to consider specific challenges that emerge in realistic environments and describe how they can be captured by tasks. These include changes of context, uncertainty, reflexive/emotional biases, cost-benefit decision-making, and balancing exploration and exploitation. Where appropriate we highlight future or current links to psychiatry. We particularly draw examples from research on clinical depression, a disorder that greatly compromises motivated behaviours in real-life, but where simpler paradigms have yielded mixed results. Finally, we highlight several paradigms that could be used to help provide new insights into the mechanisms of psychiatric disorders.

## Highlights

• Tasks incorporating ecological features provide insights into learning and decision-making.
• Distinct neural processes are recruited depending on the precise nature of the task.
• Computational modelling can help dissect component processes in complex scenarios.
• Psychiatric research may benefit from combining modelling with ecological tasks.

## 1 Introduction

Recent research in cognitive neuroscience has produced an array of paradigms and computational approaches to study facets of motivation, learning, and decision-making. While our understanding of the underlying mechanisms in the healthy brain is progressing at a fast pace, the progress made in understanding psychiatric disorders has been slow in comparison. Patients and their practitioners report very striking impairments in day-to-day learning and decision-making, yet many lab studies reveal only small differences, if any, between patients and healthy controls. Several reasons could account for this disparity including patient sample sizes [1], disease heterogeneity [[2], [3]], or that disease phenotypes cut across diagnostic criteria for different diseases [[4], [5]]. But there is another possibility explored here, namely that commonly used paradigms may not be sufficiently sensitive to the relevant features of everyday cognitive processes.

**Fig. 1:**
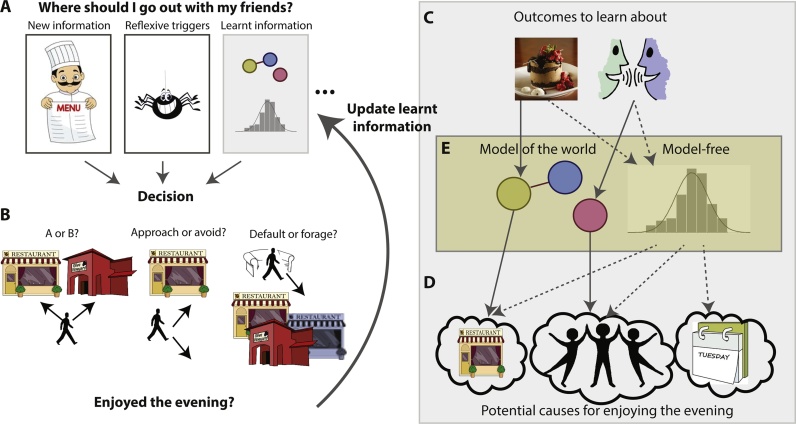
Overview of processes relevant for learning and decision-making. Many real-life situations use cognitions that can be grouped under the umbrella terms of learning and decision-making. However, examining the real-life situations more closely, we can see that there are many distinct component processes that rely on different neural substrates and can therefore be differentially affected by psychiatric disorders. A) When making decisions, we take into account different kinds of information, such as different types of rewards (e.g. money, food) or costs (e.g. delay or effort). This can be new information only available explicitly at the time of choice (e.g. reading a menu in a restaurant) or it can be learnt and recalled from past experience. Beyond these types of information that we want to take into account, other stimuli may not be relevant to the decision at hand, yet reflexively affect our judgment (e.g. seeing a spider crawl across the menu you are reading). Integrating across different kinds of information ultimately enables us to make decisions. B) In real-life situations, we make different types of decisions. Sometimes we are presented with concrete options amongst which to choose (‘A or B?’). Sometimes we have to decide whether to approach something or avoid it. Lastly, sometimes we are engaged with a behaviour (e.g. relaxing on the sofa) and need to decide whether to continue with this default or go and look for something else (e.g. decide to go into town to look for a restaurant (forage)). C) As a result of our decisions, outcomes happen (e.g. eating nice food, having a good conversation). D) However, in addition to the plausible causes for outcomes (good restaurant −> good food or friends −> enjoyable conversations), there can also be other causes present (e.g. day of the week) that are less likely to have caused the outcome. E) This multiplicity of causes and outcomes poses an attribution problem (i.e. how do you know which outcome to attribute to which cause). This can be resolved using diverse mechanisms. For example, we can either use a model of the world that tells us which outcomes and causes we should learn about and how they might relate; or we can learn in a model-free way, i.e. learn about all outcomes and causes (i.e. also about the implausible or irrelevant ones) based on how often outcomes and causes occur together. Developing tasks that can capture the ecological complexity illustrated by this example is of paramount importance for understanding the psychological and neural mechanisms underlying psychiatric disorders in real-life.

Tasks in the laboratory can be overly simplistic and not capture the sophistication and complexity of real-life situations. Alternatively, tasks can be framed in ways that are unnatural and for that reason fail to capture cognitions relevant for everyday life. To counteract these problems, basic neuroscience research has recently turned towards laboratory tasks that incorporate more ecological features ([[6], [7], [8], [9], [10]], Table 1 and Fig. 1). Here, the emphasis is on designing experiments that capture the types of processes that our brains have evolved to solve. More specifically, the idea is to identify the relevant cognitive process of interest, and to design a task, which requires the same process (and thus underlying brain networks) and thus mimics the computation identified as relevant to everyday learning and decision-making. Ecologically inspired designs do not by nature have to be more complex or be run in natural environments (there is still a balance to be struck between ecological validity and simplicity) but they require careful consideration of the processes relevant for a behaviour of interest and tasks that are adapted to precisely probe the underlying mechanisms. This review focuses on the question of whether paradigms that capture features of the cognitive processes required in real-life may also help us understand what is functionally changed in psychiatric disorders. In the majority of cases, the progress made in basic neuroscience has not yet been translated to clinical populations.

**Table 1:** A list of tasks incorporating features of learning and decision-making that are relevant in natural environments. For all of these tasks, computational models have been proposed. The reader is referred to the text and relevant papers for further details. This list is meant to highlight some important examples but it is not an exhaustive list.

Learning	
Sharot et al., Nature [252]	How strongly is the updating of people’s beliefs biased towards positive compared to negative information (‘optimism bias’)?
Behrens et al., Nat. Neurosci. [51] 2007; Mathys et al. [52] Fron Hum Neurosci., 2011	How do people flexibly adjust their speed of learning to the environment (thus providing a measure of the ability to learn that is not available when learning in stable environments)?
Jocham et al. [79], Neuron, 2016; Lee et al. [80] PLOS Biol., 2015; Leong et al. [61], Neuron, 2017	How do people attribute outcomes to causes and what are potential biases when making such attributions?
Scholl and Kolling et al., J. Neurosci. [102], 2015	How do people focus learning on what is important, ignoring irrelevant but salient reward information?
Lee et al., Neuron [97], 2014	How do people flexibly adjust, or arbitrate, between model-based and model-free learning systems?

Decision-making	
Klein-Flügge et al, PLOS Comput. Biol. [247] 2015	How do people weigh up rewards and different kinds of costs (delays or effort)?
Huys et al., PLOS Comput. Biol. [169] 2011; Guitart-Masip et al. [165], NeuroImage, 2012; Bach et al., Curr. Biol. [176], 2014	How do reflexive Pavlovian and reflective goal-directed valuation systems interact?
Eldar et al. [182] Nat. Commun., 2015	How do emotional states induced by task-irrelevant wins or losses influence subsequent evaluations of stimuli?
Wilson et al. [224] J. Exp. Psychol., 2015; Blanco et al. [29], Cognition, 2013	How do people adjust the degree of exploration and exploitation given environmental constraints?
Kolling et al. [119] Science, 2012	How do people weigh up whether to take an immediately available option or to look for alternatives elsewhere?

We will first provide an introduction to basic concepts of learning and decision-making for readers new to this field, including basic concepts of computational models of cognition. Computational modelling is essential, especially for creating more sophisticated tasks, because models can parse and quantify the performance on different sub-processes that may be recruited within the same task. In the remainder of the review, we illustrate, for both learning and decision-making, how simple paradigms have been extended to begin to capture some of the sophistication of natural environments, and how this has provided some key insights into both behaviour and brain function that cannot be gained from simpler tasks alone. Throughout, we draw examples from clinical depression to illustrate the relevance of the ecological approach. We propose that ecological tasks may help to bridge the gap between impairments seen in real-life and those reported in the laboratory. This may allow the field to advance from symptom-based to more mechanistic and quantitative diagnoses (see also [[11], [12], [13], [14], [15]]). However, we find that, while researchers have successfully begun to apply simpler paradigms to psychiatric patients, most of the more advanced and ecologically valid paradigms that we highlight have not yet been used in the context of psychiatry. One potential way forward could be to use these tasks in larger samples of patients online [[1], [16], [17]] to help identify sub-clusters within disorders but also common symptoms across disease boundaries.

## 2 Learning

### 2.1 Basic learning processes

Learning from experience is crucial for adaptive behaviour. Many decisions we make in daily life are based on values that we have learnt from experience. For example, when deciding whether to go out to meet friends at a pub, this depends on how much you have enjoyed similar experiences in the past. Studying learning holds great promise for understanding depression because it intuitively relates to many of the typical symptoms. As an illustrative example, let’s imagine a depressed patient showing social withdrawal, but on one occasion she goes out and thoroughly enjoys meeting her friends. Nevertheless, when deciding whether to go out again, she chooses not to, possibly because she did not update her belief about how enjoyable this would be. In this way, reduced learning from positive experiences could be a mechanism that maintains depression. Of course, there are other potential reasons for deciding not to go out, including for example motivational deficits, which we will consider later. But what this example highlights is how real-life complexities can be parsed into separate measurable component processes. In the following, we will first consider simple learning processes, originating from a behaviourist framework, before considering more ecological types of learning from a cognitive and computational perspective.

The simplest learning scenario, studied extensively in psychiatric research, involves the learning of associations between a single stimulus (e.g. an abstract shape) and an outcome (e.g. monetary reward). This kind of scenario directly relates to behaviourist views on behaviour and therapy [18] which have focused on different forms of conditioning (Pavlovian or operant); e.g. symptoms of anxiety are seen as a maladaptive learnt response to a particular stimulus and the treatment involves learning new responses [[19], [20], [21]].

**Fig. 2:**
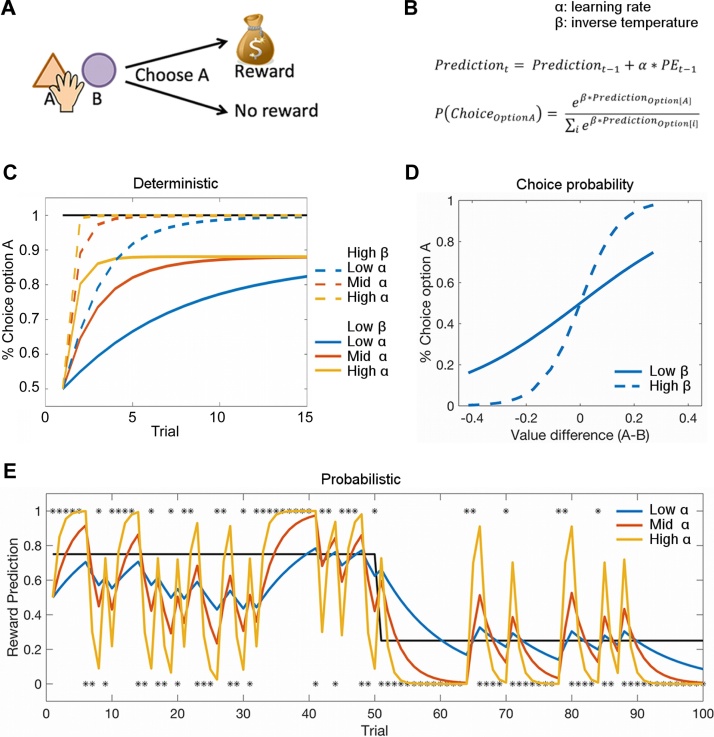
Describing learning and decision-making using computational models. A) Schematic of a task examining simple learning. On each trial, participants are presented with different options amongst which to choose. They try to choose the option that is more likely to lead to a reward. Once they have made a choice, they either receive a reward or not. Based on repeated trials, participants try to learn how likely each stimulus is to lead to reward. B) This behaviour can be described with a simple learning model. In the simplest kind of model, there are two free parameters for each person, the learning rate (α) and the inverse temperature (β): The model learns, i.e. updates its predictions, on each trial based on the difference between the outcome (reward or no reward) and the expected outcome (probability of reward), i.e. the reward prediction error (PE). The learning rate (α) determines how quickly predictions are updated based on prediction errors. Based on these predictions, the model then chooses between the options based on a softmax decision rule, i.e. the model does not always pick the option with the higher value, but only chooses that option with a certain probability (for more information see section ‘Decision-making: choice stochasticity’). How stochastic the choices are is determined by the stochasticity parameter, commonly called the ‘inverse temperature’, β (the term derives from the thermodynamics: at lower temperatures (i.e. higher ‘inverse temperature’) particles move less. Translating this to human behaviour, the higher the inverse temperature, the less random participants’ behaviour). This model can then be fitted to participants’ behaviour. This means that for each participant we determine the value for the ‘free’ parameters α and β for which the model most closely matches participants’ choices. C) The effect of different values for α and β in a deterministic task, i.e. a task in which the probability of reward is 100% for an option (and the model chooses between this option and one with a known reward probability of 50%): The higher β (dashed lines), the more likely the model is to consistently pick the option with higher value so that eventually only the better option is chosen; in contrast, for a lower β (continuous lines), the model continues to select the lower value option from time to time even once it has learnt the value of the better option. The higher α, the faster the model starts to prefer the better option. In this deterministic task, higher α is always better. D) This effect of β can also be illustrated by plotting the probability of choosing one option over the other as a function of the value difference between the two options. β is reflected in the slope of this curve, the higher β, the steeper the slope. D) The predictions learnt using different learning rates in a probabilistic task, i.e. when the stimulus only gives a reward on some trials (75% reward probability for trials 1–50 and 25% for trial 51–100, black line). Now, having a very high α (yellow) is no longer advantageous because random reward omissions (e.g. at trial 30–31) quickly pull beliefs away from the true probability. In contrast, a lower α (blue) means that beliefs are more resistant to random reward omissions. Therefore when the environment is noisy, it is more optimal to integrate information from past outcomes over a longer period, rather than relying only on the most recent observations. (For interpretation of the references to colour in this figure legend, the reader is referred to the web version of this article).

More specifically, in a typical experiment (Fig. 2A), learning is measured by presenting repeated pairings (trials) of the stimulus followed by the reward or no reward; the probability of a stimulus being followed by a reward is set by the experimenter. The measure of (clinical) interest is how participants learn these associations between stimuli and rewards, as indicated, for example, by how quickly they prefer stimuli that more likely lead to reward. To capture this learning we can use a computational model, i.e. an algorithm that simulates the processes going on in participants’ brains as they gradually learn across trials (Fig. 2B). In this particular model, learning is driven by how unexpected the outcome is (i.e., by the prediction error (PE): the difference between outcome and prediction). A large range of studies have identified PE signals in several brain areas [[22], [23], [24], [25], [26]], especially in regions receiving strong dopaminergic inputs such as the striatum. How much the PE is used (by the model or participants’ brains) to update their beliefs is determined by a free parameter in the model, called the learning rate (α). The higher the learning rate, the faster a person updates their beliefs (Fig. 2C + E). This mathematical description of the learning process as one that simply depends on how frequently a stimulus or action is paired with reward has also been coined as ‘model-free’ learning (see e.g. [27] for a review). Unlike other types of learning, it does not rely on a model of the world. However, some key features that determine whether an agent is indeed behaving in a model-free way cannot be examined by the simple task design described above. Specifically, model-free learning is not flexible. For example, you might find yourself taking your usual way to work even though you actually intended to go somewhere else today. Furthermore there is evidence that even in simple tasks, humans use other brain mechanisms, such as working memory, to supplement the model-free learning mechanism [28]. Therefore, to avoid confusion we will in the following use the term ‘simple learning tasks’, rather than ‘model-free’.

Paradigms measuring learning of these simple, behaviourist-inspired, associations have been used extensively to study learning in depression. The original hypothesis was that depression should be related to worse learning, which should be reflected in a reduced learning rate. Below we explain why this may not be the best possible hypothesis. Indeed, the behavioural results of different studies have been mixed and not consistently demonstrated learning deficits in simple learning tasks [[29], [30], [31], [32], [33], [34], [35], [36], [37], [38], [39], [40], [41]]. Changes in neural signals, by contrast, specifically a reduced PE encoding in subcortical areas including the striatum, have been reported somewhat more consistently ([[42], [43], [44]], but see also [17] for intact PE coding in a non-learning context). One cause for the variability of the behavioural findings might be that each individual study only included a small number of participants and while some findings have been brought together [45], no formal meta-analysis has been performed on the complete evidence. However, a meta-analysis [46] of a subset of these studies found no evidence for a change of the learning rate in depression. Relatedly, Gillan et al. [47] recently analysed learning in a large online sample of 1400 participants and again, found no evidence for a relationship between measures of simple (model-free) learning and questionnaire measures of depression severity.

One potential explanation for the discrepancy between real-life deficits and the apparent absence (or at best subtle nature) of basic learning deficits in depression could relate to the nature of the learning processes probed by these tasks. Using more ecological tasks could reveal learning deficits that are not apparent in simpler tasks. As we will show in more detail below (section ‘Not all value is equal‘), different types of values are processed in different brain regions. Therefore, it is possible that depression will only impact learning about some types of value but not others. Indeed, when measuring updating (i.e. learning) of beliefs about real-life events, two recent studies consistently found that depression alters learning [[48], [49]]. Healthy controls updated the beliefs of how likely negative life events were to happen to them in a biased way, i.e. they updated their beliefs more when given desirable information. By contrast, depressed patients did not show this optimism bias in learning. Another reason why ecological tasks may reveal changes in learning more clearly than simpler tasks is because more sophisticated learning mechanisms need to be recruited. In the next section, we will consider precisely such situations, namely when learning needs to be adapted to match the stability of the environment or when making causal attributions in environments with many possible causes.

### 2.2 Environmental context and adaptive learning

**Fig. 3:**
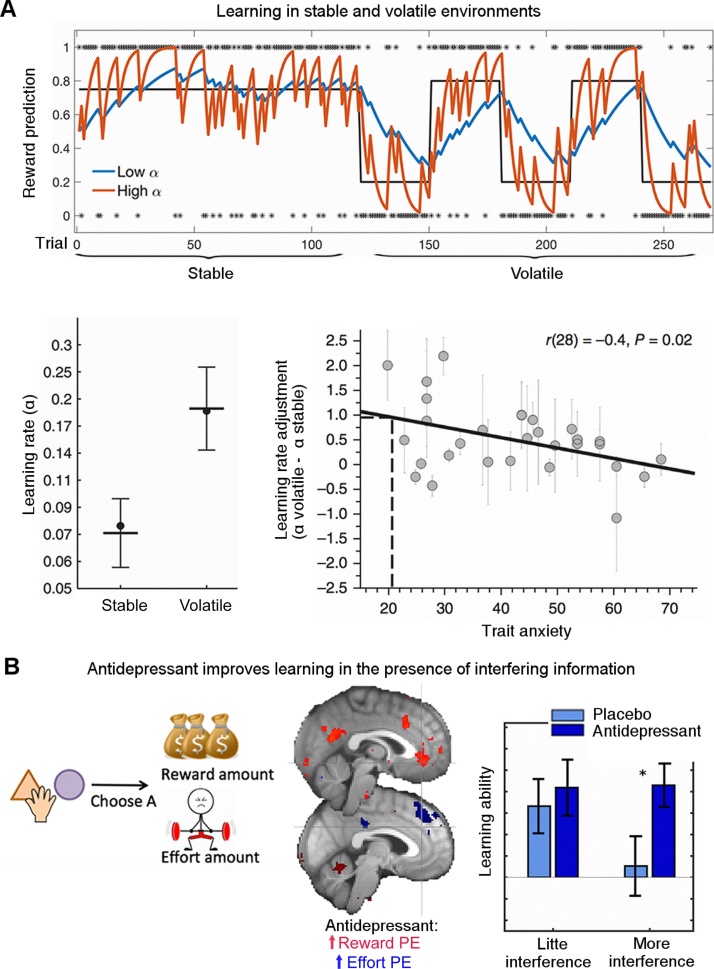
Environmental context and adaptive learning. A) Effects of different simulated learning rates (α) on learning in stable (trials 1–120) or volatile (trials 120–270) environments. In stable environments, lower α (blue) results in predictions closer to the true underlying probability of a stimulus (black) and predictions that are less affected by random reward omissions. In contrast, in unstable environments, lower α leads to predictions lagging behind the quickly changing underlying probabilities, while higher α (red) leads to predictions that track the true probabilities more closely. Behrens et al. [51] (bottom left) found that indeed human participants modulate their learning rate, learning more slowly in stable environments and faster in volatile environments. Browning et al. [53] (bottom right) found that this ability to adjust α between volatile and stable environments was related to trait anxiety with more anxious participants being less able to adjust their learning rates. B) We [56] designed an experiment to measure whether serotonergic antidepressants, that have been proposed to increase plasticity in animal models, also improve learning in humans. In the task (here simplified to the relevant features), participants repeatedly chose between two options based on their reward and effort magnitudes, which had to be learnt from experience. Neurally, the antidepressant increased learning signals, i.e. prediction errors, for both reward (left, red) and effort (right, blue). Importantly, participants had to learn different dimensions (reward and effort) simultaneously which meant that they could interfere with each other, thus making learning more difficult. Therefore, learning well could mean learning more robustly, i.e. being less affected by interference. Indeed we found that compared to placebo, antidepressants increased how well participants could learn (i.e. use prediction errors to guide future choices), when there was more interference. (For interpretation of the references to colour in this figure legend, the reader is referred to the web version of this article).

As noted earlier, in the simplest learning experiment, the speed of learning as captured by the learning rate is a measure of individual differences. Many studies focus on whether patients learn faster or slower than controls, with faster learning often being equated to better learning. However, in ecological environments, faster learning is not necessarily better learning. Instead the speed of learning should be matched to the environment ([50], Fig. 2C + E and Fig. 3A). To illustrate this, imagine you are trying to predict what mood your friend is in; if your friend generally has a stable mood, then knowing how she has felt over the last week gives you a good indication of how she feels now. In contrast, if your friend is stressed and therefore has more unstable or volatile moods, then knowing how she felt last week may not be informative. Rather, you need to find out how she felt yesterday or even an hour ago. Expressing this intuition in terms of learning rates, if the association you are trying to learn is stable, a low learning rate is advantageous but for unstable associations, i.e. those that change more quickly over time, fast updates and thus a high learning rate are more appropriate. As a computational measure of ‘the goodness’ of learning, we can then consider how well participants can adapt their learning rate to match the environment.

Tasks have been designed to measure the ability to adjust the learning rate to the environment [[51], [52]] and they have recently also been applied to psychiatric questions [[53], [54], [55]]. For example, Browning et al. [53] found that increased levels of anxiety (which is often comorbid with depression) correlated with a decreased ability to adapt to different environments. In other words, anxious individuals showed a reduced change in learning between unpredictable and changing contexts (where it makes sense to feel anxious) compared to stable contexts, which might be considered ‘safer’ (Fig. 3A). Relatedly, de Berker et al. [54] found that perceived life stress (which is a risk factor for depression) was predictive of how volatile, i.e. unstable, participants perceived an environment in a laboratory task. This relationship could suggest that life stress is the result of living in volatile or unpredictable environments. Or alternatively, the causality might be the other way around and perceiving one’s environment as more volatile than it actually is, may cause chronic stress. Of course to establish causation, future longitudinal studies are needed. Neither of those potential explanations has so far been tested in the context of depression.

The quality of learning can also be captured by parameters other than the learning rate. In environments with interfering information such as distractors or when learning about multiple things simultaneously, it might be most important to learn robustly without interference. To illustrate this, in the above example about learning how much you enjoy social encounters, imagine learning about how much you enjoy meeting your friends, while at the same time learning about how much you like the new pub and how much effort it was to get to the pub. It is clear that in this situation learning will be made more difficult by distractions that compete for processing resources. However, learning could be improved by increasing the neural strength (‘representation’) of the relevant learning signals. This would ensure that you can learn what you are intending to learn without being distracted; in other words, learning could be improved without having to change the learning rate. We measured this ability to selectively learn from the relevant feature in a study in which participants needed to learn how much monetary reward was associated with different stimuli. While doing so, there were several sources of interfering information ([56], Fig. 3B). We found that a selective serotonin re-uptake inhibitor (SSRI, citalopram) commonly prescribed to treat depression boosted the relevant neural learning signals and enabled participants to learn better in the face of interference. Of clinical relevance, finding increased neural learning signals supports the hypothesis that one way in which antidepressants act is by increasing learning or relatedly brain plasticity [57]. One future avenue might be to test whether early changes in plasticity can be predictive of treatment effects after weeks or months, thus helping to better tailor treatment to patients with potentially different combinations of symptoms of depression.

### 2.3 Attributing outcomes – the role of attention in selecting candidate causes

The learning scenarios considered so far focused on learning the strength of association between a single candidate cause and an outcome. However, in ecological situations, there is often more ambiguity about what even constitutes a potential candidate cause or more broadly a relevant stimulus (Fig. 1). For example, if you did not enjoy yourself in the company of friends, was it because your friends were overly tired, because the pub did not serve good food, because you said something wrong that upset everyone or because you wore a pink shirt? Importantly, there are distinct brain mechanisms that enable us to selectively attribute outcomes to the most appropriate causes in such situations.

The first step in this more sophisticated learning process is to narrow down the number of possible causes (from thousands) to only a few realistic ones based on ecological heuristics. This means ignoring unrealistic causes completely (e.g. wearing a pink shirt should not influence how much you enjoy your friends’ company). However, even using these heuristics, there may still be too many causes for the brain to keep track of simultaneously. Therefore, further mechanisms are needed to simplify the attribution problem. One is to selectively pay attention to only a few possible causes at a time and mentally test hypotheses about them. That means gathering evidence for or against the hypothesis that a cause predicts an outcome. This is done until the hypothesis can be confirmed or disconfirmed, and another hypothesis can be tested. There is indeed evidence that people focus attention on likely causes and that this influences how outcomes are attributed during learning [[58], [59], [60]]. Akaishi et al. [60] found, using computational modelling, that people paid attention to one hypothesis at a time and learnt selectively depending on whether this hypothesis was confirmed or not. Beyond leading to a categorical selection of potential causes, attention can also have more gradual effects (see the next section for a fuller discussion): Leong et al. [61] measured attention by tracking people’s eye movements, and found that participants deployed attention more to causes that seemed likely, and that participants were in turn more likely to attribute outcomes to the causes in their attentional focus.

There is ample evidence of attentional biases in depression, where an increased attention to negative outcomes has been reported [[62], [63]]. Relatedly, mood has been linked to the breadth of attention, i.e. to how attention is spread over different stimuli rather than focused on a single stimulus, with positive mood being linked to increased breadth of attention [[64], [65], [66]]. Interestingly, breadth of attention has also been related to noradrenaline levels, approximated by measures of pupil dilation [67]. As noradrenaline levels are known to relate to stress, this could suggest a neural substrate for how happiness or stress affect the breadth of attention. However, it remains unclear whether these attentional biases influence how patients learn to attribute outcomes to causes.

### 2.4 Attributing outcomes – neural mechanisms

**Fig. 4:**
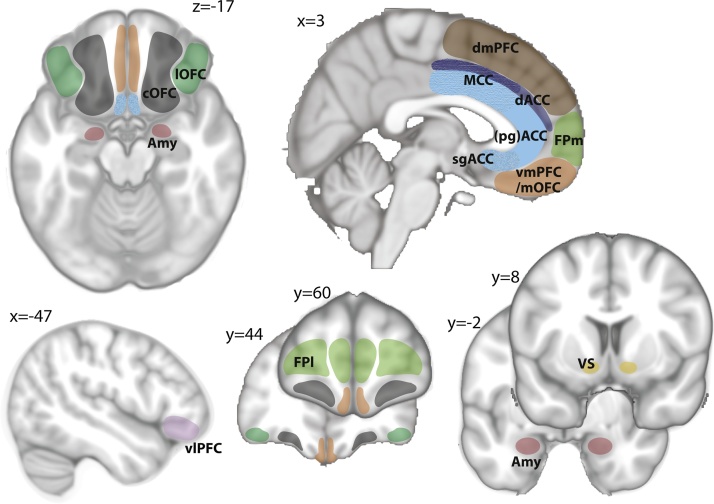
Overview of brain regions. Schematic highlighting the subcortical and prefrontal brain regions most central for the learning and decision-making processes discussed in this review. The anterior cingulate cortex (ACC) is shown in shades of blue with the dorsal sulcal portion dACC/ACCs in dark blue and the ventral gyral portion ACCg in light blue. The posterior aspect of ACCg and ACCs is also referred to as midcingulate corext (MCC) [[236], [237]], the aspect curving around the genu of the corpus callosum as perigenual ACC (pgACC) and the most ventral portion as subgenual ACC (sgACC). The frontal pole (FP, green) spans a large area of cortex and can be subdivided into its medial (FPm) and lateral (FPl) portion. Note that these are the abbreviations used throughout the review but there is not always consistency in the literature (for example, what we refer to as central orbitofrontal cortex (cOFC) has sometimes been referred to as medial OFC (mOFC) as well). (vmPFC = ventro-medial prefrontal cortex; dmPFC = dorso-medial PFC (includes pre-SMA); lOFC = lateral OFC; vlPFC = ventrolateral prefrontal cortex; VS = ventral striatum; Amy = Amygdala). For more detailed maps see [[237], [238], [239], [240], [241]]. (For interpretation of the references to colour in this figure legend, the reader is referred to the web version of this article).

Having narrowed down the number of possible causes you are considering at any one time, how does the brain correctly attribute outcomes to their underlying causes? Specific component processes rely on different specialized brain areas (see Fig. 4 for the anatomical location of the brain areas discussed in the following sections). As those component processes could lead to behavioural changes in unique ways we will consider them individually below. The concept of component processes is of particular relevance when considering the heterogeneity of psychiatric disorders because certain processes could be affected by a disorder but others left intact; and different subtypes of a disorder could affect different component processes.

**Fig. 5:**
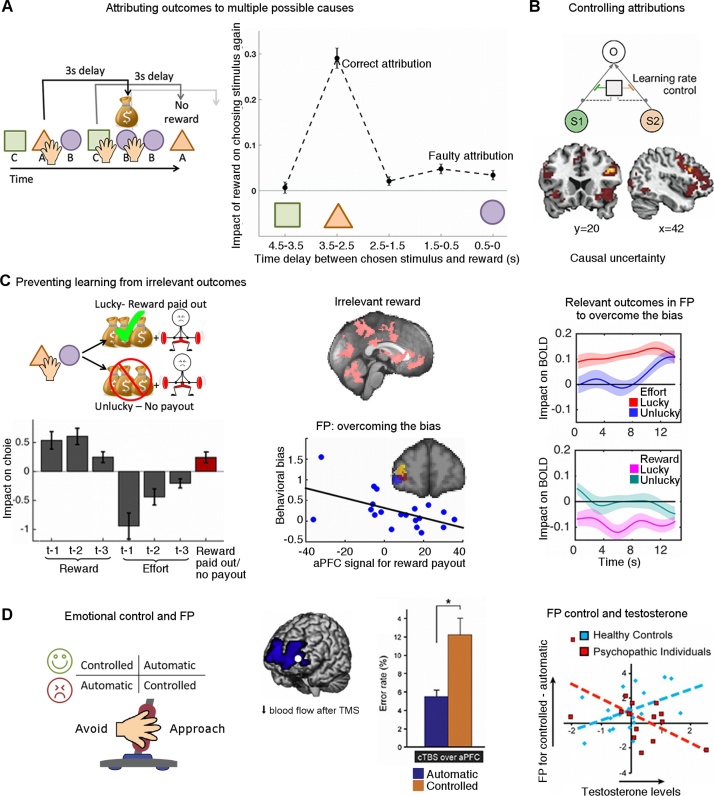
Different mechanisms for making attributions. A) Jocham et al. [79] designed an experiment to assess how well people can attribute outcomes to correct causes or instead make faulty attributions. In the task (simplified here), participants were presented with a continuous stream of stimuli, each appearing on the screen for 1.5 s. For each stimulus, participants could either choose it for a small cost (hand symbol) or ignore it. If participants selected a stimulus it led to a reward (with a certain probability) after a fixed 3 s delay. This meant that in between having selected a stimulus (e.g. orange triangle) and receiving its reward, other stimuli appeared on the screen. Thus, the reward could potentially be misattributed to other stimuli that did not cause it. Behavioural results (right) showed that participants were mostly attributing outcomes to the correct causes: they were most likely to select a stimulus again that had appeared about three seconds before the reward. However, they also misattributed outcomes to causes that occurred just before the outcome (1.5 s to 0s). B) Lee et al. [80] designed an experiment to measure to what degree outcomes were associated to different plausible causes, or in other words how high the learning rate was for each cue. They found that how the learning rate was split amongst different cues depended on the causal uncertainty: the more participants were uncertain about whether a cue had caused an outcome, the higher the learning rate for that cue. Neurally, they found that ventrolateral prefrontal cortex represented this causal uncertainty. C) We [102] designed an experiment to assess how the brain avoids misattributing salient, yet irrelevant, outcomes. In the task (simplified here), participants repeatedly chose between two symbols based on how much reward and effort was associated with each stimulus. In addition, there was also salient, but irrelevant, outcomes not dependent on participants’ choices, namely whether the amount of reward from the current trial would be paid out as monetary reward (green tick) or not (red cross). Behavioural analyses (bottom left) revealed that participants’ choices were mostly guided by the relevant dimensions (i.e. reward and effort amounts on the past three trials (t-1 to t-3)). However, they also misattributed the irrelevant outcomes: they were biased to choose an option again if it had led to a reward payout (red bar). This bias was potentially present because many areas in the brain were sensitive to the irrelevant reward outcome (pink, top middle column). We found several signals in frontal pole (FP) that might suggest that this area plays a role in overcoming the bias: FP carried a signal for the irrelevant reward payout and the people who had a stronger FP signal were less biased (bottom middle column). Additionally FP increased its representation of the information to be learnt (amount of reward and effort outcome) when reward was paid out thus potentially helping to overcome the bias (right column). D) Volman et al. [104] tested whether FP also plays a role for overcoming automatic emotional biases during approach-avoidance decisions in social contexts. On each trial, participants had to make an approach or avoid decision (i.e. move a joystick towards or away from themselves) after a very brief presentation (100 ms) of a happy or an angry face. Half of the actions were reflexive/automatic (i.e. approach-happy and avoid-angry) and the other half controlled (i.e. approach-angry and avoid-happy). Disruptive transcranial magnetic stimulation (TMS) over FP changed blood flow in bilateral FP. At the same time, TMS also selectively increased the rate of errors in the controlled condition. Interestingly, in a separate study [106], the same authors found that in psychopaths with particularly high testosterone levels, activity in FP in the controlled compared to the automatic condition was decreased. This suggests that maybe they are less able to control the impact of reflexive emotional information during rule-driven behaviour. (For interpretation of the references to colour in this figure legend, the reader is referred to the web version of this article).

Distributed neural networks can process and keep online the relevant pieces of information or relevant dimensions of an outcome that determine its value: First, the reward *identity* needs to be processed. This could be the broader category of reward (e.g., a social reinforcer or food item) or its specific characteristics (e.g., salty or sweet). For instance, when processing food rewards, outcome identity is encoded in a region of prefrontal cortex, namely the posterior and medial region of OFC [[68], [69]]. Second, it is important to keep in *memory* what potential causes or stimuli are currently relevant, when those do not appear at the same time as the outcome. To date, our knowledge of such representations is limited, but a recent study found evidence for stimulus-specific memory traces in the same sensory areas that initially processed the potential causes in the experiment [70]. Third, a representation of the *strength of the association* between the candidate causes and outcomes to be updated needs to be stored; there is evidence that the hippocampal memory systems [71] and more lateral OFC (lOFC) [69] keep track of this information. To make correct attributions, all these different mechanisms need to work together. Different lines of evidence suggest that integration of these different pieces of information happens in lOFC. When this area is lesioned, monkeys [[72], [73], [74]] and humans [75] no longer attribute outcomes to the correct causes. Additionally, fMRI studies have shown that during the learning process, lOFC is active when specific attributions are made [[71], [76], [77], [78], [79]]. Using a more complex design with many possible causes (see Fig. 5A for detailed explanation), Jocham et al. [79] found that people in whom lOFC is more active during learning are better at making correct attributions.

Knowing which causes and outcomes to consider generally, the next question is how much of the outcome to attribute to each cause. To return to the example of figuring out why you enjoy socializing, imagine one day there is a new person present. If everything else is the same, and you feel different, it would seem most likely that this change in your feelings is attributable to the new person. In other words, a good heuristic is that surprising outcomes are more likely to be due to causes that you are uncertain about or know less about (e.g. because they are new) than by ones you are familiar with. We can quantify this intuition in mathematical terms as uncertainty about how a cause and an outcome are related. That people indeed use uncertainty to guide attributions has been confirmed in a recent study using a novel design ([80], Fig. 5B). Participants had to learn to attribute outcomes to causes with different causal uncertainty. Neurally, the computation of uncertainty was linked to a region in the prefrontal cortex, namely the ventrolateral PFC (vlPFC). It is interesting to note the potential parallels between this gradual effect of uncertainty on learning and the gradual effects of attention [61] and environmental volatility [[51], [52]] discussed above. These *quantitative* effects whereby a potential cause is given a stronger or weaker weight in predicting an outcome stand in contrast to more categorical selection effects where some potential causes are completely disregarded so that outcomes will not at all be attributed to them.

Interestingly, completely independently from the above mentioned line of research, faulty attributions have been proposed as a key mechanism in the development of depression [[81], [82]]. Questionnaire-based studies have found that patients with depression or healthy people who later go on to develop depression are more likely to attribute negative events to themselves rather than external causes, compared to healthy controls [[81], [82], [83], [84], [85], [86], [87]]. However, results from experimental studies have been less clear-cut ([[88], [89], [90], [91], [92]] and [93] for a review). It is therefore currently not possible to conclude that the attribution of outcomes to their underlying causes is a process that is generally impaired in depression. The application of new and more ecological paradigms, such as the ones described here and in the next section, could help to shed light on how the diverse mechanisms are affected in depression.

### 2.5 Attributing outcomes – different strategies for different situations

The mechanisms for making attributions described above work well when agents have an accurate model of the world, i.e. when they know what the plausible causes are and what outcomes are important. However, in the real world, it may sometimes not be possible to have a precise model and one’s model may also be entirely wrong. For example, if you do not even consider the possibility that the cause of your friends liking you is because you are a nice person (and instead only consider external causes such as them being polite), you can never learn about your niceness. A solution to this problem might be to have concurrent learning mechanisms, some of which indiscriminately learn about all possible causes that are present, without filtering out unlikely causes.

Indeed, studies in which the lOFC – the region we described above as being important for attributing causes based on a model – is lesioned show that non-human primates can still learn, but that they use a different strategy [[72], [73]]. Specifically, control monkeys associated outcomes most strongly with stimuli that actually caused them (e.g. a stimulus preceding an outcome in the same trial). In contrast, after lesions, monkeys associated outcomes with stimuli that were temporally proximal to the outcome even if they could not have caused the outcome (e.g. stimuli from previous or subsequent trials).

While such a strategy of learning by temporal proximity is not optimal if you have a model of the task, it is a good additional strategy in many natural environments where it is often less clear which cause is going to produce which outcome and when. Indeed, Jocham et al. [79] found evidence that such a learning mechanism exists by changing the task structure from one in which outcomes could be clearly associated with previous causes (as shown in Fig. 3B; and which humans solved using lOFC) to one in which it was not clear when in the past causes for outcomes had occurred (i.e. when participants could not use a model of the task; not shown). In the latter situation, humans changed their learning and now associated outcomes with choices at varying times in the past. This type of imprecise learning has been termed ‘spread-of-reward’ and linked to the amygdala [[78], [79]].

### 2.6 Selecting the appropriate learning mechanisms

We have considered how more complex learning may depend on different kinds of brain mechanisms all operating together at the same time. But learning with several independent brain systems in parallel raises a new issue: How do you decide which one should guide choices? For example, one system could tell you to choose stimulus A, while the other one might favour stimulus B.

While this has not yet been studied for attributional learning, it has been studied in the context of model-based and model-free learning. We will not consider the task in detail − it has been covered in some excellent reviews [[27], [94], [95], [96]]. In brief, in this task, there are also different ways in which participants can learn, relying on a complex model of the task (‘model-based’), or on simpler mechanisms (‘model-free’). Lee et al. [97] proposed that one way to decide which system should be used to drive choices would be to monitor how reliable the knowledge of either system is. To examine this, they manipulated how well different parts of their task could be solved by each of the systems. They found that the signature of this process of assessing the reliability of each system was associated with activity in a distinct area of prefrontal cortex, namely the vlPFC and frontal pole. It may be important to note that while this specific task and related versions of it [[98], [99]] measures one form of what is referred to as ‘model-based’ learning, there are many other ways in which learning can be ‘model-based’ or in other words, rely on a model of the task. As described above, for example, using a model of the world to know what stimuli are potentially relevant, using a model of how quickly the environment is changing to adapt the learning rate, using uncertainty to adapt the learning rate, learning from fictive feed-back [[100], [101]], or finding out which learning mechanism is appropriate (as described here). This is important because these very distinct forms of learning that are not ‘model-free’ rely on different brain mechanisms and are therefore likely affected differently by different psychiatric conditions.

Unlike situations where there is ambiguity about which learning mechanism is appropriate, there are situations where the model of the world is clear and model-free learning is not appropriate. For example, when meeting friends in the bar, it is clear that whether or not you like the music should not inform your judgment of how much you like your friends. However, if the music is emotionally salient (strongly driving the model-free learning mechanism relying on temporal proximity discussed above), it may be hard to ignore it. Model-free learning in this context means that the pleasant feeling caused by the music spills over to your judgements of your friends because the two events co-occurred in time. In contrast, if you have an accurate model of the world (i.e. you know that the enjoyment of the music cannot be caused by your friends), you should not make this misattribution. How can the brain deal with this kind of situation? We tested this in an experiment in which participants had to learn to predict how much reward was associated with different stimuli [102] (Fig. 5C). In that experiment there were also irrelevant, yet salient, rewards (like the music in the bar). We found that indeed, people were biased by these rewards and misattributed them to the stimuli, even though the optimal behaviour would have been to completely ignore them. Such a bias may exist because many brain areas are sensitive to reward [103]. On the other hand, we also found a network centred on the frontal pole which encoded signals that suggested it was trying to compensate for this bias. The frontal pole increased its representation of the relevant information when the salient information needed to be ignored, while also producing a signal driving behaviour to overcome the bias. In other words, when an irrelevant reward experience biased participants to choose one option, this brain area would produce a signal to change participants’ preference towards the other option. These findings are in agreement with studies of emotional control during decision-making ([[104], [105], [106]], Fig. 5D) and with findings showing that lesions of this area and nearby areas in humans lead to misattributions of outcomes to irrelevant dimensions [107]. Thus, in situations in which it is clear what brain mechanism should guide behaviour, the brain not only selects the most relevant mechanism, but actually over-writes, or counter-acts, learning from other inappropriate mechanisms. It will be interesting to investigate in the future how psychiatric disorders affect the abilities to flexibly arbitrate between different learning systems and to suppress inappropriate learning mechanisms.

### 2.7 Building beliefs about the world

So far, we have focused on diverse mechanisms for learning the *reward value* of stimuli. Another important aspect in naturalistic environments is of course learning about the *structure* of the world or, in other words, learning a cognitive map. More specifically, a cognitive map has information about how different states in the environment relate to each other and how different stimuli should be processed in different states. For example, you can think of different ways to interpret a friend ignoring you, depending on your internal model: you could think ‘my friend does not like me’, based on a model, or a negative self-view, made up of a whole range of connected statements such as ‘I am worthless’ or ‘people don’t like me’. Or you could think ‘my friend didn’t see me’, based on a different model that includes statements such as ‘how other people act often has more to do with their state than with me’. The states, with their specific cognitive content, in this kind of model, directly relate to the concept of ‘schemas’ in psychiatry, which have been used to explain aspects of depression. For example, Beck’s theory of depression proposes negative schemas, i.e. sets of beliefs about the self and the world and how they relate to each other, as a problem in depression [[108], [109], [110]]. In this theory, people possess different schemas (e.g. ‘I’m great’ and at the same time ‘I’m a failure’) and they can become active at different times. In depression for example, stressors are thought to activate negative schemas that then shape perception and action and thus trigger a depressive episode. A person who had been depressed in the past may possess the same schemas but they would lie dormant.

Neurophysiologically, the vmPFC/mOFC system (Fig. 4) has been related to hidden states and inferential models, i.e. schemas that determine how to process stimuli, or a cognitive map that can be searched through or employed in simulations [[111], [112], [113], [114], [115]]. For example, Schuck et al. [115] designed a task in which participants needed to make decisions about stimuli based on a model of the task, i.e. knowing the different task states that they had previously learnt (e.g. is feature A or feature B of the stimulus relevant for your decision?). To make the decisions, they needed to infer which (hidden) task state was currently relevant. This information was represented in vmPFC/mOFC, thus supporting the view that this region has a mental model/map. As an aside, we also note that this is not the only type of mental model and other research has highlighted the dorsal anterior cingulate cortex in the context of learning the structure of the world and using this knowledge to explore the environment [[116], [117], [118], [119], [120], [121], [122], [123], [124], [125], [126]]; but this is beyond the scope of this review.

While there are intuitive links to be drawn between mental maps of the sort studied in these experiments and the schemas invoked in theories of depression, the kind of paradigm described above has not yet been used to test whether depressed and healthy controls differ, for example, in how flexibly they can shift between different task schemas or whether they have biases towards certain kinds of schemas.

## 3 Valuation and decision-making

### 3.1 Using new information and integrating multiple sources of value

Thus far in this review, we have focused on different learning mechanisms and on how they might influence our choices. Yet, in everyday life, to guide behaviour we often need to integrate *learnt* information with *newly perceived* information (Fig. 1, left-hand side). To illustrate this, in the example about enjoying the company of your friends, imagine deciding whether to go out for dinner with them. This not only depends on how much you have recently enjoyed their company, but it may also depend on what the restaurant has on the menu today, the cost of the food or your appetite. Here we will first focus on the valuation of new information, and the integration of costs; the decision process will be covered later in this section. Studying valuation processes is of great importance for psychiatry, as patients commonly have motivational problems in specific contexts, possibly because they do not value them appropriately. For instance, lack of energy or fatigue is a core symptom of depression [127] and this may impact on choices requiring physical effort, e.g. deciding to spend energy to get to the restaurant, but not other types of choices. Furthermore, different brain regions process different types of value information. This, in turn, may mean that different groups of patients, with different underlying pathology (e.g for depression see [128]), experience differing types and degrees of impairment depending on the nature of the valuation problem probed by a given task.

### 3.2 Not all value is equal

**Fig. 6:**
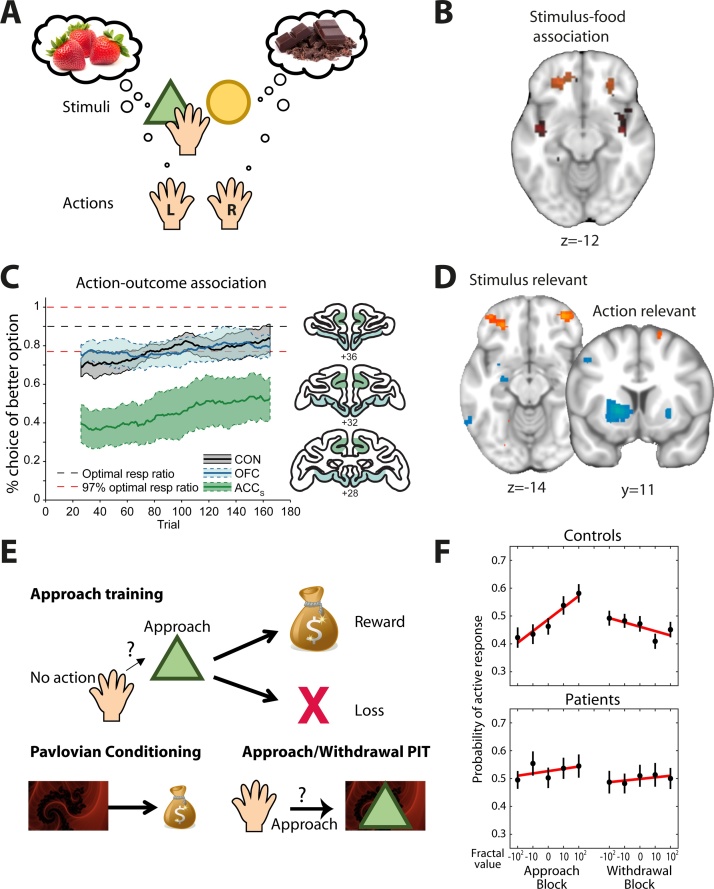
Different brain valuation systems. **A**) Illustration of a simple binary choice task that assesses valuation processes when assigning value to stimuli (e.g. abstract shape, top) or actions (e.g., right or left hand, eye or joystick movement, bottom). In some paradigms information changes over time but it is also possible to vary the properties of the visual stimulus to signal the expected outcome which requires evaluating new information. For example, the colour and quantity of symbols might denote the juice type and quantity of juice as in [68]. **B**) Encoding associations between stimuli and reward relies on central orbito-frontal cortex (orange; red is irrelevant here). Here this was shown using repetition suppression [69] which provides a way to study neural representations using human neuroimaging [242]. **C**) In contrast, the anterior cingulate cortex (ACC) is critical for using action-outcome associations to guide choice as shown here using lesions in macaque monkeys (shaded areas show lesions in OFC and ACC sulcus respectively; adapted from [117] and [138]). **D**) When both actions and stimuli are relevant for guiding choices, interactions with several valuation networks take place (interactions with parietal cortex when actions are relevant (blue: striatum) or stimuli are relevant (orange: OFC)). Adapted from [148]. **E**) Sometimes, new information elicits reflexive changes in value. In the Pavlovian-Instrumental-Transfer (PIT) task [169], participants first learn whether or not to approach visual stimuli (triangle) to obtain reward and avoid a punishment (in separate blocks, participants had to learn whether or not to actively withdraw from a stimulus to get a reward and avoid punishment; not shown). In the second stage, they passively view several Pavlovian stimuli (fractal) followed by tones predictive of wins or losses (−100, −10, 0 10, 100). In the test phase, the measure of interest is how the Pavlovian stimulus (fractal) affects decisions about whether or not to approach/withdraw from the previously learnt instrumental stimuli (triangle). No outcomes are shown in this phase. **F**) The optimal behaviour would be to ignore the incidental Pavlovian stimuli. However, the pattern usually observed in healthy controls is that in approach blocks (top left), participants are more likely to approach the instrumental stimulus if the Pavlovian stimulus in the background has a higher positive value than if it has a negative value. The opposite behaviour is seen in avoid/withdrawal blocks (top right). This rudimentary response was absent in depressed patients (bottom) (adapted from [175]). (For interpretation of the references to colour in this figure legend, the reader is referred to the web version of this article).

It might be tempting to think of value as being one singular currency that predicts a person’s preference. However, there is now ample evidence to show that the brain contains multiple valuation systems [[129], [130]]. Broadly speaking, the value of an outcome will be represented in the brain regions that process the information the value is constructed from (Fig. 6A–D). For example, evaluating a food item involves processing its taste, visual appearance and smell, and these sensory inputs converge in the central orbitofrontal cortex (cOFC, Fig. 4) [131], a region critical for processing the value of food items. In experiments, a reward is often predicted by a visual cue displayed on the screen (a Starbuck’s sign might make you think of coffee) and evidence from lesion, recording and neuroimaging studies demonstrates cOFC’s essential role in representing multiple dimensions of stimuli such as the associated food type or value (Fig. 6B) [[68], [69], [132], [133], [134], [135], [136], [137], [138], [139], [140]]. In contrast, an outcome can also be predicted as a result of performing a certain action, rather than from a visual or sensory cue. For example, finding out whether your best friend is coming along for dinner involves picking up the phone, and thus assigning value to the result of an action plan. Typically, in tasks investigating action value representations, different actions, e.g. eye movements or joystick movements lead to different amounts or types of reward. Evaluating rewards that are tied to actions involves a different network of brain regions to the one described above, including the dorsal striatum and anterior cingulate cortex (ACC) [[141], [142], [143], [144]]. Lesions to ACC impair choices guided by action values (but not stimulus values) in macaques and human patients (Fig. 6C) [[138], [145]]. This is consistent with the connectivity profile of ACC; it has much weaker sensory inputs than OFC but more direct projections to premotor and motor cortices [[146], [147]]. Compellingly, when multiple attributes (e.g., actions *and* cues) are relevant for evaluating a choice option, interactions between several of the brain regions we have discussed take place (Fig. 6D) [[76], [148]]. Thus, *where* in the brain new information is evaluated will depend on *what* the value is assigned to (a food outcome, an abstract cue, an action etc.). This is worth bearing in mind when designing tasks for specific patient populations, as they could manifest problems of value corresponding to the functional circuit that is affected by their pathology. One type of value we have not touched upon here but which may have great relevance for psychiatric disorders is the value of social information. Many real-life situations involve several individuals and thus, it is important for tasks to manipulate social context and the type of social information that needs to be processed. This is not covered here due to space constraints (but see [[149], [150], [151]]).

So far, in depression, a range of valuation deficits have been reported but overall the conclusions have been quite mixed [[152], [153]]. Basic tests such as the sucrose liking test [[154], [155]] show no difference between healthy controls and patients suffering from depression. Similarly, ratings of cartoon images with emotional content were found to be unchanged in depression [156] and a recent study deploying a computational model to assess valuation of gambles in the absence of learning found no difference between patients suffering from depression and controls [157]. By contrast, other studies have found reactions to emotional stimuli to appear blunted in depression. For example, sad movies did not seem to trigger the same increase in sad feelings in depressed patients as in controls [158]. While it was originally thought that patients suffering from depression are overly sensitive to negative feedback and show a catastrophic response to failure [[159], [160]], a more recent study suggested instead that there might be a diminished sensitivity to emotionally negative information which leads to less adjustments in subsequent behaviour, meaning unlike controls, patients would not correct their performance as much after error feedback [161]. Evidence from a meta-analysis including a wide range of measures (self-report, behavioural, physiological) suggest that this reduced response to emotional stimuli is present in both the positive and negative domain [162]. Still, overall results have been mixed which could be related to several reasons. These include the particular valuation system probed by a task, the large variety of biotypes of depression [128], or alternatively, the way participants ‘report’ value. For example, studies relying on reflective valuation processes (e.g. when participants give ratings or make deliberate choices [[156], [157]]) may recruit different brain systems than those relying on automatic valuation processes (e.g. approaching or avoiding; see next section). All of these are open questions that will need to be addressed by future work.

### 3.3 Reflective versus automatic valuation

There are not only different types of value for e.g. actions and stimuli, but also differences in terms of cognitive accessibility [163]. The valuation processes discussed thus far are largely ‘reflective’ in the sense that they involve thinking about and imagining possible outcomes, as well as considering which cues are relevant for achieving the outcomes. The goal of this reflective process is to act in a goal-directed way. However, sometimes a new piece of information elicits a more automatic, and thus less controlled, change in value. If, for example, a spider unexpectedly walked across the restaurant table when you are out with your friends, you may not enjoy your food as much. Considering this situation from the perspective of reflective (or model-based) reasoning, what has gone wrong here is that – assuming the spider disappeared again and there is no action you currently need to take – the stimulus (the spider) is irrelevant to your current task (eating) and should therefore not influence your choices. While this situation illustrates that it is not always appropriate for values to become active and influence behaviour, these types of automatic influences on behaviour are ubiquitous, raising the question as to why this would be. As considered in depth in previous reviews [[94], [96], [163], [164]], the advantage of automatic/reflexive systems is that they are computationally efficient, and therefore available more quickly, which can be important in many real-life situations. For example, if a bear is approaching you, running away quickly out of fear is more adaptive than carefully considering how likely it is that the bear will hurt you. One way in which automatic influences on valuation can be studied is in tasks measuring approach and avoidance tendencies either without ([104], Fig. 5D) or with a learning component [165]. These approach/avoid decisions recruit a network centred on amygdala and subgenual ACC (sgACC) [166], a prefrontal region with a high density of amygdala inputs [167], for review, see [168]. Reflexive influences on behaviour can also be measured using Pavlovian-Instrumental-Transfer (PIT) tasks where an incidental appetitive or aversive Pavlovian cue that is irrelevant to the task at hand (e.g., the spider) increases the likelihood to approach or avoid another stimulus (e.g., your food), respectively [169][e.g., 169] (Fig. 6E). In both types of tasks, there is a strong link between valuation and action and the effect of the incidental cue is measured in terms of action (approach/avoid). Interestingly, the brain networks driving the influence of Pavlovian cues on value and behaviour are primarily phylogenetically older structures, in particular striatum and amygdala [[170], [171], [172], [173]]. Automatic valuation signals have also been identified in neo-cortical regions such as vmPFC (e.g. [[102], [174]] (Fig. 5)). Altogether, this suggests that the strength of a reflexive bias (or value) is encoded separately from the action that it biases.

Reflexive valuation mechanisms and their interaction with more reflective systems are of particular interest for psychiatry, and they might relate to aspects of cognitive therapy. Huys et al. [175] used a PIT task to examine how Pavlovian cues influence valuation in depression (Fig. 6F). In healthy participants, a positive incidental cue promoted approach and a negative incidental cue promoted avoid responses, respectively. However, this rudimentary response was absent in depressed people. As the authors pointed out, one potential consequence of this might be that in depression approaching a positive or avoiding a stressful situation might rely on more computationally expensive (‘reflective’) neural mechanisms and thus feel more effortful. If this is the case then it may begin to explain some of the symptoms of depression such as anhedonia and increased exposure to stressful live-events. Future studies could build on this work to test the idea that in depression Pavlovian approach-avoid tendencies are reduced, using for example a recent task proposed by Bach and colleagues [176] where Pavlovian impulses for approaching and avoiding produced helpful behavioural responses rather than unhelpful behavioural biases as in PIT tasks. Overall, it seems that in depression the interaction between a more automatic and a more reflective valuation system are off-balance, with a reduced influence of more automatic valuation mechanisms [175]. In line with this, lowering central serotonin levels using acute tryptophan depletion had similar effects, reducing approach/avoid behaviour, yet only in the aversive domain [177]. And when deciding whether to approach or avoid an outcome, stimulation in a ventral zone of sgACC induced depressive phenotypes (negative biases) and altered approach/avoid type choices but not choices between two options [166]. All of these findings are particularly interesting given depression is associated with structural and functional brain changes in the amygdala, ventral striatum, and sgACC [[43], [161], [178], [179], [180], [181]]. In other words, both neurophysiological and behavioural evidence point towards reduced use of reflexive value and increased reliance on reflective computations, which could together reduce motivation and lead to rumination. In related work, Eldar et al. found that people with high mood instability (a risk factor for bipolar disorder), valued monetary rewards differently as a result of an incidental event (large win or loss) that affected their current mood [[182], [183]]. Specifically, participants had to learn how likely different slot machines (i.e. stimuli) were to give rewards. After learning about half of the slot machines, participants experienced a large win or loss in a wheel-of-fortune draw, which affected participants’ mood. However, whether they won or lost was not related to the next set of slot machines that participants subsequently went on to learn about. Despite being aware of this, participants with high mood instability behaved as if the slot machines after a large reward were better than the ones after a large loss. This is another example of automatic processes interfering with goal-directed processes.

### 3.4 Integrating costs and benefits

**Fig. 7:**
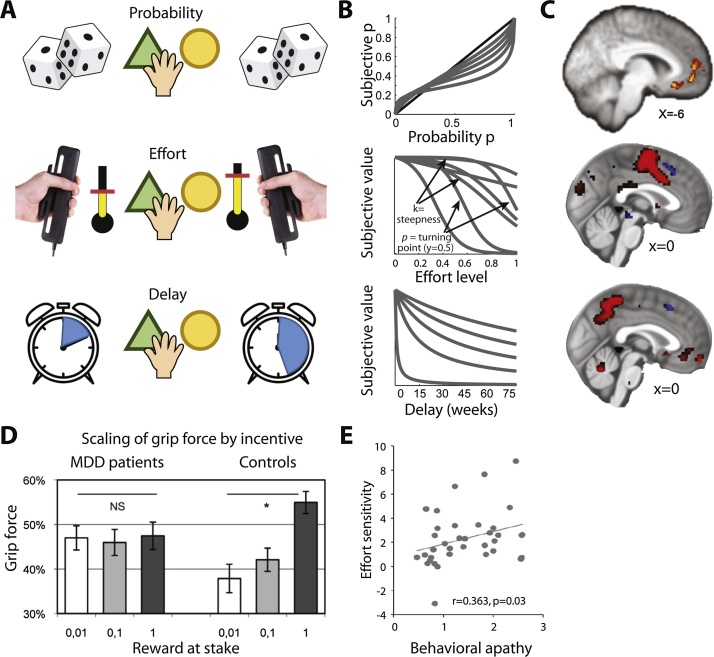
Mechanisms for integrating costs into value. **A**) Illustration of binary choice tasks involving different types of costs. In all cases, choices are made between two options, A and B, associated with varying quantities of a monetary or food reward (displayed or pre-learnt). Importantly, the reward comes at a cost which could be the ‘risk’ associated with winning the reward (varying probabilities, top), the physical effort that needs to be exerted to obtain the reward (e.g., grip force, middle), or the delay before which the reward will be received (bottom). **B**) To model the integration of costs and benefits into subjective value and capture an individual’s discount preference – e.g., how much the subjective value of an option with a fixed reward decreases as a function of different cost levels − simple behavioural models are used. The parameter(s) fitted for each individual explain how the value of reward decreases with increasing costs. *Top*: for probability, prospect theory provides the standard model accounting for the over-weighting of small and under-weighting of large probabilities that most individuals exhibit [[243], [244], [245]]; *middle*: for effort discounting, the best fit is achieved using an initially concave function, for example an inverse sigmoidal (shown here) or parabolic/quadratic function (not shown) [[246], [247]]; *bottom:* for delay discounting, a convex hyperbolic model is appropriate [[248], [249]]. The different shapes of the discounting functions suggest that different types of costs affect choices differently: for example the concave shape for effort discounting meant that people care not much about whether they have to make a small effort or no effort, but they care more when the choice is between a small and a medium effort. In contrast, for delays, people will care about whether a reward will be paid out immediately or only in an hour, but they care less about whether reward is received in three weeks, or three weeks plus an hour. Having a model of subjective value provides several critical advantages. First, it provides sensitivity to inter-individual differences. Second, it can be used to capture the influence of cost in situations involving more than one relevant variable. Third, fitted parameters can be used to examine relationships with other behavioural or clinical markers. Finally, neuroimaging data can be explained in ways that would otherwise not be possible, for example by looking at the representation of value difference as a marker for choice. **C**) Three exemplar studies looking at the encoding of value (i.e. the difference in value between the chosen and unchosen option; red: activation; blue: deactivation) for the three different types of costs (top: probability; middle: effort; bottom: delay). This highlights distinct networks depending on the type of cost (red), while the inverse contrast is consistently encoded in dmPFC (blue). Adapted from [[101], [185], [189]]. **D**) The effort (here grip force) exerted to get a reward scales with expected reward (x axis) in controls but not depressed patients. Adapted from [199]. **E**) Participants performed a task where they accept or reject a reward given the required effort. A model captured each individual’s effort sensitivity, or in other words, how much weight they placed on the effort in their choices. This parameter was directly related to an individual’s apathy trait with higher apathy relating to higher effort sensitivity. Adapted from [197]. (For interpretation of the references to colour in this figure legend, the reader is referred to the web version of this article).

So far we have focussed on the valuation of rewarding outcomes, or benefits, and aversive outcomes, such as losses. However, in natural environments, valuation processes frequently entail an integration of costs and benefits. The types of costs typically encountered include temporal delays (e.g., having to wait for your friend to pick you up), motor costs (e.g., having to walk to the bus stop), and risks (e.g., getting caught in a traffic jam). Tasks looking at how a given cost affects the evaluation of a rewarding outcome usually offer participants two options that are each associated with different levels of cost and reward (Fig. 7A). Alternatively, sometimes only one option varies in reward and cost and another option stays constant across trials (‘default’) (for effort costs see e.g. [[184], [185], [186]]). Simple behavioural models can be used to capture an individual’s propensity to be influenced by a given type of cost and the most commonly used examples of such ‘discounting functions’ are shown in Fig. 7B.

The brain networks recruited to encode and integrate different types of costs again differ depending on the type of information that needs to be processed. For instance, the vmPFC encodes information about subjective value when probability (i.e., risk) and magnitude need to be integrated to evaluate an option (Fig. 7C, top) [[101], [187]]. When evaluating whether a reward is worth waiting for, a similar but sometimes slightly more dorsal and posterior perigenual ACC (pgACC/vmPFC) region encodes subjective values [[188], [189], [190]] (Fig. 7C, bottom). By contrast, when physical energy is necessary to obtain an outcome, more dorsal regions in ACC/MCC, with more direct projections to premotor and motor cortices, are essential for encoding the integrated cost-benefit value (Fig. 7C, centre) [[102], [117], [185], [191], [192], [193], [194], [195], [196], [197]].

Valuation deficits in depression may possibly be found most consistently when physical effort is involved. In other words, when there is a need to energize behaviour through self-motivated actions and thus express how much it is worth working for an outcome. For instance, going out with your friends requires mobilizing some energy to get to the restaurant. Unlike in some of the domains of learning and valuation covered above, there is converging evidence that this process might be altered in depression, and many symptoms of depression, such as lack of energy, fatigue or decreased engagement in activities, relate to this. For instance, healthy controls produce more effort for higher reward incentives, but depressed individuals show overall reduced levels of effort production [198] and no scaling of effort with expected reward size ([[156], [199]], Fig. 7D). This nicely dissociates liking an outcome from being willing to produce motivated behaviour to obtain it. Changes in the willingness to exert effort for reward are also seen in patients suffering from apathy ([[200], [201]], Fig. 7E), and apathy has been found to be strongly associated with symptoms of depression in a large online cohort [47]. Several things about the existing work on effort in depression are striking. First, it remains quite unclear where exactly the deficit lies: in the mobilization of effort *per se*, the integration of rewards and costs, or some aspect of the reward itself. Second, it is unclear how specific this deficit is to physical, as opposed to other types of costs, e.g. mental effort or temporal delay. Finally, and most importantly, the effect sizes reported in laboratory studies are small compared to the deficits observed in real-life (e.g., for energy loss see [202]). This suggests that experimental paradigms need to better capture the complexity of real-life situations, and combine this with careful computational modelling. For example, many real-life decisions are about whether to continue with a default behaviour or make an effort to look for alternatives (e.g. deciding to stop relaxing on the sofa to go out, see Fig. 1B). Instead, the situation that is often encountered in laboratory tasks is to face two concrete options (with explicit rewards and efforts) to choose between (see also Fig. 9B).

### 3.5 Integration of different sources of value: the role of different neurotransmitter systems

Another critical question for decision-making is how much to rely on different types of value, for instance information we have learnt over time in contrast to newly perceived information. For example, if a new cook has started to work in the restaurant that your friends are taking you to, your previous experience should influence your choice to go out with them less. In addition to asking whether different brain areas encode different types of value, there has been interest in whether different neurotransmitters and neuromodulators influence *how* this integration occurs. This is especially important for psychiatry as many treatments are drug-based and involve particular pharmacological targets.

**Fig. 8:**
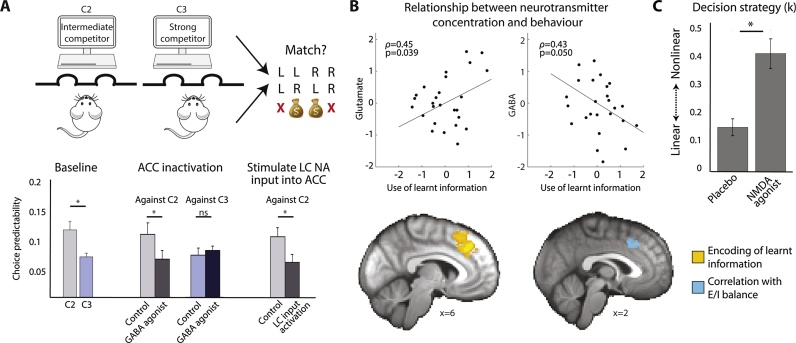
Molecular mechanisms for integrating information. **A**) Top: Rats are playing against either an intermediate (C2) or a strong competitor (C3) who is trying to predict their choice between two food ports. Animals are only rewarded on trials where the computer does not match their choice. The difference between the competitors is that the strong competitor is better able to detect any patterns in the animal’s choices and exploit them. In other words, if there are any statistical regularities, i.e. if the rats’ behaviour is predictable, the competitor will use this information to prevent the rats from getting rewards. Bottom: Playing against a stronger competitor leads to more random choices (decreased choice predictability). Inactivation of anterior cingulate cortex (ACC) using a GABA agonist produces increasingly random choices in rats playing against C2, but not in the rats playing against C3 who already exhibit strongly random choice behaviour. This effect is mediated via noradrenergic (NA) input from the locus coeruleus (LC) onto ACC because the same effect is observed when LC inputs to ACC are stimulated pharmacologically or optogenetically. This suggests that the level of NA in the ACC controls the balance between using a model (trying to predict the opponent) and random choices. Adapted from [122]. **B**) Magnetic mesonance spectroscopy (MRS) was used to examine the balance of excitation (glutamate) and inhibition (GABA) in the dACC when choices relied on learnt and explicitly cued information. Top: A model parameter that captured how much participants relied on learnt relative to new information showed a positive relationship with Glutamate and a negative relationship with GABA. Bottom: dACC encoded the information to be learnt and this signal increased as a function of the E/I balance in the region. Thus, consistent with [122], dACC controls how much learnt information influences behaviour and this might be achieved by regulating its E/I-balance. Adapted from [126]. **C**) In a similar task, a partial NMDA agonist made participants rely more strongly on an optimal non-linear strategy for combining different pieces of information to calculate integrated value (here reward probability * magnitude). Choices in the placebo group were guided predominantly by the simpler linear computation (here reward probability + magnitude). Adapted from [209]. This suggests that NMDA receptors play a role in non-linear integration of information.

The neurotransmitter noradrenaline (NA), is a good candidate for regulating how much to rely on learnt information (e.g., given past experience, how likely am I to enjoy the time with my friends). In rats, increasing the influence of NA on ACC activity and thus decreasing ACC activity, caused animals to abandon prior knowledge, whereas silencing NA had the opposite effect, making them more reliant on learnt information ([122], Fig. 8A). In humans, magnetic resonance spectroscopy (MRS) can be used to measure the concentration of the two main excitatory and inhibitory neurotransmitters, glutamate and GABA. Using MRS, we were able to show that the balance of excitation and inhibition (E-I balance) in dACC regulates how much choices rely on learnt versus newly perceived value information ([126], Fig. 8B). Higher levels of glutamate and lower levels of GABA were associated with both increased strength of the value to be learnt in dACC and increased use of learnt information over new information when making choices. Taken together, these results suggest that the dACC plays a crucial role in allowing information that has been learnt to influence behaviour (see also section ‘Building beliefs about the world’), and that, on a molecular level, this may be realised by regulating the E-I balance which in turn is possible via the noradrenergic system. This is interesting because the E-I balance can be altered in acute stress [203] and when manipulating serotonin levels [204], as would typically be done with SSRIs in depression. Some reports also suggests that glutamate/glutamine and GABA levels, specifically in ACC, are reduced in depression [[205], [206]] but there is mixed evidence regarding this possibility [[207], [208]]. It is therefore plausible that such neurochemical changes could affect how different aspects of value are integrated in these patients. Indeed, we have recently made the observation that patients with dysphoria use learnt information less, compared to new information, when making decisions (unpublished).

In addition to asking whether learnt (or other) value information should be weighed more or less strongly when making a choice, it is also important to think about how information is combined and how this might be influenced by neurotransmitter levels. In other words, how does the brain implement linear or non-linear combinations for forming an estimate of integrated subjective value from, e.g. reward and probability (see discounting curves for combining costs and benefits in Fig. 7)? We tested this in another study ([209], Fig. 8C), and quantified the degree participants relied on a linear or non-linear strategy for combining values with a computational model. We found that a partial NMDA agonist made participants rely more strongly on the optimal non-linear strategy while choices in the placebo group were guided predominantly by the simpler linear computation. This is in agreement with previous findings that NMDA receptors facilitate non-linear integration of information in e.g., multisensory integration [210]. Our data suggests that this role extends to the value domain. While in this study, the integration happened between learnt and newly cued information, it is plausible that a similar mechanism of integration might be recruited when several explicit cues need to be integrated non-linearly to compute value – e.g., the delay and size of reward or effort and size of reward.

### 3.6 Decision-making: choice stochasticity

The previous sections have focussed on mechanisms of learning, the valuation of new information and integration of different pieces of information. In this section, we focus on the decision or selection process itself. Indecisiveness, or a greater difficulty in making choices, is a core symptom of depression (e.g., [211]). Depressed individuals tend to avoid making decisions, show maladaptive decision-making, and enhanced stress levels when making choices [[212], [213], [214], [215], [216]].

It seems straightforward that once the integrated values of different choice options have been computed, for example the values assigned to different options for spending your evening, the option with the highest subjective value should consistently be chosen. This is not, however, what is usually observed. For example, in a simple situation where one option gives reward 80% of the time, and a second option only 20% of the time, participant’s choices generally match these probabilities in that option 1 is chosen 80% of the time. However, to maximise reward, option 1 should be chosen 100% of the time. This raises the question of where this randomness, or stochasticity, in the observed choices comes from. Many different possible sources might contribute, such as the desire to explore other options in case their value has changed; uncertainty about underlying value estimates and the optimal way of integrating different aspects of value (covered above); priors that skew values in one direction; and mistakes, for example due to distraction, tiredness, carelessness. Below we will first describe how choice stochasticity is usually accounted for in computational models before discussing why it may provide an advantage in ecological settings.

**Fig. 9:**
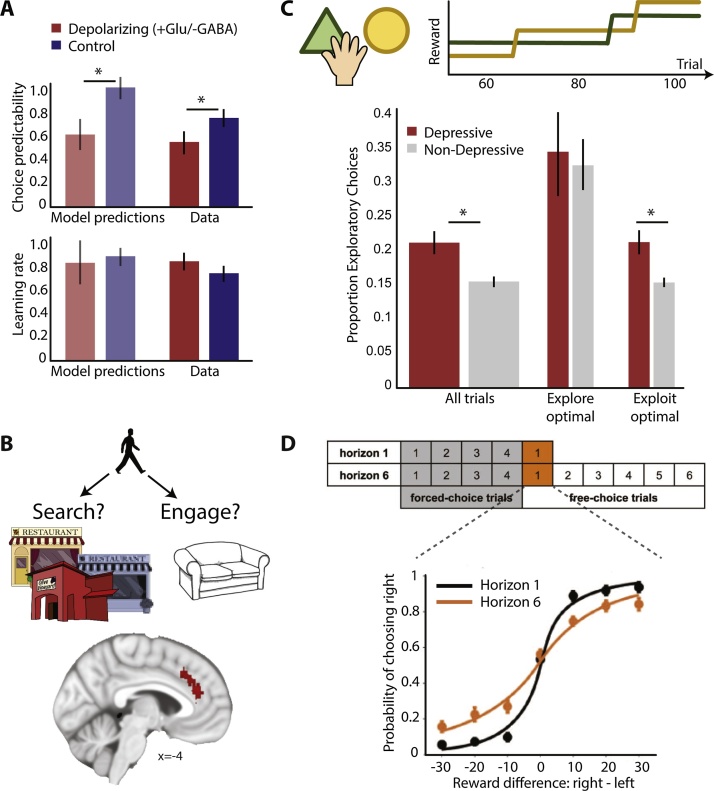
Neural mechanisms underlying exploration. **A**) Participants performed a binary choice task in which the probability of reward had to be learnt while reward size was cued on the screen. Transcranial direct current stimulation (tDCS) targeted the vmPFC, a region critical for comparing the values of options when reward magnitude and probability need to be integrated. Anodal tDCS is thought to depolarize the underlying pyramidal neurons thus causing a shift in the E/I balance towards more excitation [[250], [251]]. tDCS led to more exploration (smaller softmax inverse temperature) in agreement with predictions generated using a biophysical model (top). In contrast, learning (measured as learning rate in the model) was not changed (bottom). This dissociation would not have been possible without a learning model and suggests that the degree of exploration in this task is regulated via the balance of excitation and inhibition in vmPFC. Adapted from [218]. **B**) Example study in which choices were not framed as being between options A and B but between whether to engage with a currently present option (or in other words a ‘default’) or to explore the environment (i.e. search in the environment for other opportunities). Studying the neural circuits underlying this ecological type of choice revealed the dACC as the key region representing the value of searching/exploring. Adapted from [8]. **C**) Choices between two options are made and one of the options is consistently better until the alternative increase in value (‘jumps up’) at an unpredictable time (Leapfrog task). Exploratory choices (i.e., checking whether the alternative has changed) should become more frequent the more time has passed since the last ‘jump’. Students with symptoms of depression were overall more exploratory (or in other words less consistent in picking the option with higher value, i.e. their behaviour was more random). However, most importantly, a closer look at when the exploration happened showed that depressed participants did not explore more on trials when exploring was advantageous (to check for ‘jumps’) but instead they explored more at times when exploiting would have been the optimal behaviour. Adapted from [29]. **D**) Wilson et al. [224] designed a task to measure whether humans adjust how much they explored, depending on how useful it was to do so. In each game of the task, participants were presented with two slot machines. In the first four trials of each game they could not choose themselves which slot machine to play, but instead the computer selected an option for them (‘forced choice trials’). Participants were only shown how much they won from the machine they played, not how much they could have won from the alternative. The key manipulation was that after these forced choices participants were given either a single trial (‘horizon 1′) or six trials (‘horizon 6′) on which they could choose freely between the two slot machines (‘free choice trials’). In horizon 6 compared to horizon 1, it was thus more valuable to gather information about which slot machine was better because more choices were left where this information could be exploited. And indeed (bottom panel in D), participants were more likely, at the first free choice, to explore (and thus select the slot machine that had so far been less valuable) in horizon 6 compared to horizon 1 (see also to Fig. 2D).

When using computational models to explain participants’ choices in tasks, it is important to account for choice stochasticity. This is typically done by fitting a parameter, referred to as the softmax inverse temperature, for each participant which given the value of an option, provides an estimate of the likelihood of choosing this option (Fig. 2B–D). Work using MRS in humans has shown that the degree of stochasticity relates to the E-I balance in the ventromedial prefrontal cortex, a brain region with a role in comparing choice option values [217]. Relatedly, causal manipulation of the E-I balance in this region (using depolarizing transcranial direct current stimulation (tDCS)) leads to increasingly stochastic choices (Fig. 9A, [218]). Both of these results make sense in the context of current models whereby the competition between several choice options is resolved via mutual inhibition [[219], [220]]. An increased concentration of GABA, and decreased concentration of glutamate, would imply increased levels of inhibition, a slower more precise comparison process and thus less random choices [217]. By contrast, increased levels of excitation will make the comparison process converge faster, thus generating more stochastic choices [218].

Interestingly, despite a relatively good neuro-computational understanding of stochasticity in decisions and decision-making problems being central to depression with obvious real-life problems associated with it, there are few studies showing changes in choice randomness in the lab, and most do not dissociate valuation from choice stochasticity in depression. Some reports find increased reaction times in simple binary choices in depression without changes in accuracy [221]. Changed reaction times could point at changed decision making as current decision models make predictions about both choice and reaction time patterns. This effect could, for example, be due to blunted valuation, in essence, making the choice harder because values seem more similar. However, if this is the case then decision-making should also be less accurate in depressed patients but this is not the case. Alternatively, if valuation is unperturbed, increased reaction times can be achieved via increased inhibition/reduced excitation in the comparison process, but such changes would predict depression-related improvements in accuracy, which are not observed either. Interestingly, Huys et al.’s meta-analysis [46] reported reduced reward sensitivity in depression by fitting computational parameters to a reward-learning tasks. Because in the model, reward sensitivity could not be dissociated from stochasticity per se, this provides evidence for increased decision randomness in depression. In contrast, another recent study [157] that used a decision-making task, based only on explicitly shown, rather than learnt values, found no effect of depression on choice stochasticity. Overall it thus remains unclear whether the decision deficits reported in depression are truly deficits in the comparison process. However, maybe instead of more fine-grained measurements of choices and reaction times, we should start thinking differently about the reasons why a decision-maker might not always choose the best option in the first place. Such choices may not simply reflect stochasticity in the decision mechanism itself but instead they may reflect the operation of distinct goal-directed processes. In the next section we focus particularly on mechanisms for exploration.

### 3.7 Choice randomness in ecological environments enables exploration

It is important to consider why humans and animals would have developed a natural tendency to produce stochastic choices. What seems like suboptimal or noisy behaviour in simple laboratory settings turns out to actually provide an advantageous behavioural strategy in ecological settings, emphasizing once more the need for ecologically valid task designs. For example, the probability matching behaviour described earlier is the same as that found when an optimal Bayesian model is supplied with ecologically valid prior beliefs, such as a prior that events happening close in time are not unrelated [222]. Another aspect of natural environments is that they are not usually stable in terms of the outcomes predicted for a given choice. In an environment where friendships develop or the cook can change, it is optimal to occasionally explore the value of alternatives (e.g., how to spend your evening) to maximise overall gain over the longer term [223]. Few studies thus far have dissociated random choices from targeted exploration. However, Wilson and colleagues designed a task (‘Horizon task’; Fig. 9D) that allowed dissociating random choice from purposeful exploration and showed that humans explored more when it was more useful to do so [224]. Furthermore, directed but not random exploration was affected by transcranial magnetic stimulation (TMS) over right frontopolar cortex [225] suggesting different neural systems support the two types of exploration. Other work in which choices were encoded in a frame of exploring versus exploiting showed that dACC holds a representation of the value of exploring the environment ([[119], [226]], Fig. 9B). Not only does activity in dACC change when exploratory choices are made but when the outcomes of exploratory choices are evaluated [[120], [227], [228]]. Choice stochasticity, or in other words unpredictable choices, can also confer a critical advantage when faced with an opponent who is trying to predict our behaviour, and inactivation of rat ACC increases the degree of randomness ([[122], [229]], Fig. 8A). Designing tasks that make it possible to determine the degree to which choice stochasticity is strategic, whether as a way of exploring and refining the model of the world or to confuse a competitor may be critical for understanding changes in decision-making in psychiatric disorders.

To our knowledge, only one study so far has distinguished targeted from random exploration in depression; this work does suggest that depressed patients may show altered exploratory behaviour. Blanco et al., [230] used the ‘Leapfrog task’ where the reward obtained from two options can only ever increase over time (Fig. 9C). The inferior option can jump up at unpredictable times, so that on a given trial the choice is between exploiting what is thought to be the best option, and exploring whether the previously inferior option might have exceeded it. In this task, exploration should be structured, with an increased likelihood of exploration as time since the last exploratory trial passes. Indeed, the majority of control participants’ behaviour resembled such a pattern, while half of the patients suffering from depression (and particularly those with the highest levels of depression) lacked specifically this exploration strategy. Interestingly, overall, depressed patients produced more choices that could be seen as exploratory, but on trials when they should have exploited the current option. Thus, this study nicely illustrates the importance of ecological designs and of using behavioural models to better capture people’s behaviour, and suggests that goal-directed exploration might be altered in depression.

### 3.8 Perils of the ecological approach and possible solutions

While we have tried to outline the various advantages of probing cognitive processes with tasks that incorporate ecological features, there are of course also some caveats that researchers need to be aware of. One concern is the increased complexity of ecological tasks, which may mean that a single behaviour (e.g. pressing a button) is now influenced by several different factors. This stands in contrast to more classical designs where all but one factor are kept constant. The question that arises in ecological tasks then is how to dissociate different cognitive processes and define their unique contributions. Here we propose that this problem can be addressed by using computational models of the processes recruited by a given task (see for example [[231], [232]] for an introduction). As one illustrative example, we can consider a study on reinforcement learning in depression by Huys et al. [46] (see also section on ‘basic learning processes’). The authors showed that by using a computational model, a simple behaviour (i.e. choices on each trial that could be summarized as proportion correct) could be parsed into separate components, namely the speed of learning (learning rate) and the choice stochasticity (Fig. 2). Then, the next question that arises is about which model(s) to use. Choosing the right model is critical and the usual approach would be to construct several plausible models (e.g. in the example from Huys et al., models that consider an absence of reward as a punishment) and then select the model that best describes the data using model comparison [233]. Of course, model comparisons are limited to selecting the best model out of the models that are being considered and thus may rely on researchers’ subjective choices. We can increase our confidence that we have indeed found a ‘good enough’ model by including model simulations, i.e. by letting the different models that have been fit to behaviour perform the same task as the participants. Then, we can check that the simulated behaviour captures key features of interest found in the behaviour of real participants [234] and that this is *only* the case for the model of interest, not for alternative models [235]. In general, the problems of complexity and model selection can be managed by increasing the sophistication of tasks gradually, by building on previous work, and by carefully defining the key cognition of interest in the first place.

Another source of concern might be the interpretation of findings obtained using more ecological tasks. As we have tried to illustrate, umbrella terms such as ‘learning’ often capture a range of different cognitive processes. Importantly, these processes can be described very precisely with separate parameters in a computational model, and thus computational modelling usually aids the interpretation of findings in more sophisticated tasks. For example finding that anxiety changes how well the learning rate is adjusted to match the environment should not be interpreted as a ‘general learning deficit’ (see section ‘Environmental context and adaptive learning‘).

Given these caveats, it is clear that great care needs to be taken when designing and analysing experiments with ecological features and computational models. Fortunately, the field of computational neuroscience is moving towards open science, which we believe will accelerate the progress that can be made. It will enable direct access to resources, such as the code for a particular model and source data that can greatly aid replication and re-analysis.

## 4 Conclusions

In this review, we have argued that our understanding of learning and decision-making, in particular in the context of psychiatric disorders, could greatly benefit from considering tasks that probe more ecological features. Indeed, studying learning and decision-making in ways that capture aspects of naturalistic environments has started to reveal distinct cognitive processes that rely on different neural substrates, which would not be recruited in simple tasks. This diversity also highlights that generic umbrella terms such as ‘learning’ encompass a large variety of distinct and only partly overlapping neural processes. Thus, symptoms of psychiatric disorders may only emerge when probing the specific mechanisms that are also recruited in naturalistic scenarios relevant for the disorder under consideration. It is therefore essential to use tasks that incorporate ecological features and that are specifically designed to target the process of interest (Table 1). We have highlighted how computational modelling in combination with more ecological tasks can allow the dissociation of different behavioural processes and the characterization of different neural systems. We hope this approach will enable better characterization of the diversity of an individual’s behaviour (in other words creating computational ‘fingerprints’ of a person’s cognitive abilities) and by extension enable mapping of subgroups of patients and symptoms more reliably. While psychiatric research has begun to apply some of those computational ideas, many fruitful avenues for future research remain. Ultimately, the results of these studies could help to build new unifying theories of psychiatric disorders that can be translated into patient treatment.

## Funding

Jacqueline Scholl is funded by an MRC Skills Development fellowship (MR/N014448/1) and Miriam Klein-Flügge is funded by a Sir Henry Wellcome fellowship (103184/Z/13/Z).

## References

[1] Gillan C.M., Daw N.D. (2016). Taking psychiatry research online. Neuron.

[2] Chekroud A.M., Gueorguieva R., Krumholz H.M., Trivedi M.H., Krystal J.H., McCarthy G. (2017). Reevaluating the efficacy and predictability of antidepressant treatments: a symptom clustering approach. JAMA Psychiatry.

[3] Fried E. (2017). Moving forward: how depression heterogeneity hinders progress in treatment and research. Expert Rev. Neurother..

[4] Borsboom D., Cramer A.O.J. (2013). Network analysis an integrative approach to the structure of psychopathology. Annu. Rev. Clin. Psychol..

[5] Wang X.-J., Krystal J.H. (2014). Computational psychiatry. Neuron.

[6] Pearson J.M., Watson K.K., Platt M.L. (2014). Decision making the neuroethological turn. Neuron.

[7] Calhoun A.J., Hayden B.Y. (2015). The foraging brain. Neuroeconomics.

[8] Kolling N., Behrens T., Wittmann M., Rushworth M. (2016). Multiple signals in anterior cingulate cortex. Neurobiol. Cogn. Behav..

[9] Addicott M.A., Pearson J.M., Sweitzer M.M., Barack D.L., Platt M.L. (2017). A primer on foraging and the explore/exploit trade-off for psychiatry research. Neuropsychopharmacology.

[10] Bach D.R., Dayan P. (2017). Algorithms for survival: a comparative perspective on emotions. Nat. Rev. Neurosci..

[11] Corlett P.R., Fletcher P.C. (2014). Computational psychiatry: a Rosetta Stone linking the brain to mental illness. Lancet Psychiatry.

[12] Adams R.A., Huys Q.J.M., Roiser J.P. (2015). Computational Psychiatry: towards a mathematically informed understanding of mental illness. J. Neurol. Neurosurg. Am. Psychiatry.

[13] Dayan P., Dolan R.J., Friston K.J., Montague P.R. (2015). Taming the shrewdness of neural function: methodological challenges in computational psychiatry. Neuroeconomics.

[14] Huys Q.J.M., Maia T.V., Frank M.J. (2016). Computational psychiatry as a bridge from neuroscience to clinical applications. Nat. Neurosci..

[15] Moutoussis M., Eldar E., Dolan R.J. (2016). Building a new field of computational psychiatry. Biol. Psychiatry.

[16] Brown H.R., Zeidman P., Smittenaar P., Adams R.A., McNab F., Rutledge R.B., Dolan R.J. (2014). Crowdsourcing for cognitive science? The utility of smartphones. PLoS One.

[17] Rutledge R.B., Moutoussis M., Smittenaar P., Zeidman P., Taylor T., Hrynkiewicz L., Lam J., Skandali N., Siegel J.Z., Ousdal O.T., Prabhu G., Dayan P., Fonagy P., Dolan R.J. (2017). Association of neural and emotional impacts of reward prediction errors with major depression. JAMA Psychiatry.

[18] Beck A.T. (1970). Cognitive therapy: nature and relation to behavior therapy. Behav. Ther..

[19] Wolpe J. (1954). Reciprocal inhibition as the main basis of psychotherapeutic effects. AMA Arch. Neurol. Psychiatry.

[20] Lazarus A.A. (1958). New methods in psychotherapy: a case study. South Afr. Med. J..

[21] Lazarus A.A. (1977). Has behavior therapy outlived its usefulness?. Am. Psychol..

[22] Schultz W., Dayan P., Montague P.R. (1997). A neural substrate of prediction and reward. Science.

[23] O’Doherty J.P., Dayan P., Friston K., Critchley H., Dolan R.J. (2003). Temporal difference models and reward-related learning in the human brain. Neuron.

[24] Bayer H.M., Glimcher P.W. (2005). Midbrain dopamine neurons encode a quantitative reward prediction error signal. Neuron.

[25] Rutledge R.B., Dean M., Caplin A., Glimcher P.W. (2010). Testing the reward prediction error hypothesis with an axiomatic model. J. Neurosci..

[26] Garrison J., Erdeniz B., Done J. (2013). Prediction error in reinforcement learning: a meta-analysis of neuroimaging studies. Neurosci. Biobehav. Rev..

[27] Dayan P., Berridge K.C. (2014). Model-based and model-free pavlovian reward learning: revaluation, revision, and revelation. Cogn. Affect. Behav. Neurosci..

[28] Collins A.G.E., Frank M.J. (2012). How much of reinforcement learning is working memory, not reinforcement learning? A behavioral, computational, and neurogenetic analysis: working memory in reinforcement learning. Eur. J. Neurosci..

[29] Blanco N.J., Otto A.R., Maddox W.T., Beevers C.G., Love B.C. (2013). The influence of depression symptoms on exploratory decision-making. Cognition.

[30] Chase H.W., Frank M.J., Michael A., Bullmore E.T., Sahakian B.J., Robbins T.W. (2010). Approach and avoidance learning in patients with major depression and healthy controls: relation to anhedonia. Psychol. Med..

[31] Cella M., Dymond S., Cooper A. (2010). Impaired flexible decision-making in major depressive disorder. J. Affect. Disord..

[32] Herzallah M.M., Moustafa A.A., Natsheh J.Y., Abdellatif S.M., Taha M.B., Tayem Y.I., Sehwail M.A., Amleh I., Petrides G., Myers C.E., Gluck M.A. (2013). Learning from negative feedback in patients with major depressive disorder is attenuated by SSRI antidepressants. Front. Integr. Neurosci..

[33] Kunisato Y., Okamoto Y., Ueda K., Onoda K., Okada G., Yoshimura S., Suzuki S., Samejima K., Yamawaki S. (2012). Effects of depression on reward-based decision making and variability of action in probabilistic learning. J. Behav. Ther. Exp. Psychiatry.

[34] Mueller E.M., Pechtel P., Cohen A.L., Douglas S.R., Pizzagalli D.A. (2015). Potentiated processing of negative feedback in depression is attenuated by anhedonia: research Article: anhedonia and Feedback Processing in MDD. Depress. Anxiety.

[35] Must A., Szabó Z., Bódi N., Szász A., Janka Z., Kéri S. (2006). Sensitivity to reward and punishment and the prefrontal cortex in major depression. J. Affect. Disord..

[36] Pechtel P., Dutra S.J., Goetz E.L., Pizzagalli D.A. (2013). Blunted reward responsiveness in remitted depression. J. Psychiatr. Res..

[37] Remijnse P.L., Nielen M.M.A., vanBalkom A.J.L.M., Hendriks G.-J., Hoogendijk W.J., Uylings H.B.M., Veltman D.J. (2009). Differential frontal-striatal and paralimbic activity during reversal learning in major depressive disorder and obsessive-compulsive disorder. Psychol. Med..

[38] Taylor Tavares J.V., Clark L., Furey M.L., Williams G.B., Sahakian B.J., Drevets W.C. (2008). Neural basis of abnormal response to negative feedback in unmedicated mood disorders. Neuroimage.

[39] Thoma P., Norra C., Juckel G., Suchan B., Bellebaum C. (2015). Performance monitoring and empathy during active and observational learning in patients with major depression. Biol. Psychol..

[40] Vrieze E., Pizzagalli D.A., Demyttenaere K., Hompes T., Sienaert P., de Boer P., Schmidt M., Claes S. (2013). Reduced reward learning predicts outcome in major depressive disorder. Biol. Psychiatry.

[41] Msetfi R.M., Murphy R.A., Kornbrot D.E. (2012). Dysphoric mood states are related to sensitivity to temporal changes in contingency. Front. Psychol..

[42] Kumar P., Waiter G., Ahearn T., Milders M., Reid I., Steele J.D. (2008). Abnormal temporal difference reward-learning signals in major depression. Brain.

[43] Gradin V.B., Kumar P., Waiter G., Ahearn T., Stickle C., Milders M., Reid I., Hall J., Steele J.D. (2011). Expected value and prediction error abnormalities in depression and schizophrenia. Brain.

[44] Robinson O.J., Cools R., Carlisi C.O., Sahakian B.J., Drevets W.C. (2012). Ventral striatum response during reward and punishment reversal learning in unmedicated major depressive disorder. Am. J. Psychiatry.

[45] Chen C., Takahashi T., Nakagawa S., Inoue T., Kusumi I. (2015). Reinforcement learning in depression: a review of computational research. Neurosci. Biobehav. Rev..

[46] Huys Q.J., Pizzagalli D.A., Bogdan R., Dayan P. (2013). Mapping anhedonia onto reinforcement learning: a behavioural meta-analysis. Biol. Mood Anxiety Disord..

[47] Gillan C.M., Kosinski M., Whelan R., Phelps E.A., Daw N.D. (2016). Characterizing a psychiatric symptom dimension related to deficits in goal-directed control. Elife.

[48] Garrett N., Sharot T., Faulkner P., Korn C.W., Roiser J.P., Dolan R.J. (2014). Losing the rose tinted glasses: neural substrates of unbiased belief updating in depression. Front. Hum. Neurosci..

[49] Korn C.W., Sharot T., Walter H., Heekeren H.R., Dolan R.J. (2014). Depression is related to an absence of optimistically biased belief updating about future life events. Psychol. Med..

[50] O’Reilly J.X. (2013). Making Predictions in a Changing World—Inference, Uncertainty, and Learning.

[51] Behrens T.E.J., Woolrich M.W., Walton M.E., Rushworth M.F.S. (2007). Learning the value of information in an uncertain world. Nat. Neurosci..

[52] Mathys C., Daunizeau J., Friston K.J., Stephan K.E. (2011). A Bayesian foundation for individual learning under uncertainty. Front. Hum. Neurosci..

[53] Browning M., Behrens T.E., Jocham G., O’reilly J.X., Bishop S.J. (2015). Anxious individuals have difficulty learning the causal statistics of aversive environments. Nat. Neurosci..

[54] De Berker A.O., Rutledge R.B., Mathys C., Marshall L., Cross G.F., Dolan R.J., Bestmann S. (2016). Computations of uncertainty mediate acute stress responses in humans. Nat. Commun..

[55] Marshall L., Mathys C., Ruge D., de Berker A.O., Dayan P., Stephan K.E., Bestmann S. (2016). Pharmacological fingerprints of contextual uncertainty. PLoS Biol..

[56] Scholl J., Kolling N., Nelissen N., Browning M., Rushworth M.F., Harmer C.J. (2017). Beyond negative valence: 2-week administration of a serotonergic antidepressant enhances both reward and effort learning signals. PLoS Biol..

[57] Harmer C.J., Duman R.S., Cowen P.J. (2017). How do antidepressants work? New perspectives for refining future treatment approaches. Lancet Psychiatry.

[58] Wilson R.C., Niv Y. (2012). Inferring relevance in a changing world. Front. Hum. Neurosci..

[59] Niv Y., Daniel R., Geana A., Gershman S.J., Leong Y.C., Radulescu A., Wilson R.C. (2015). Reinforcement learning in multidimensional environments relies on attention mechanisms. J. Neurosci..

[60] Akaishi R., Kolling N., Brown J.W., Rushworth M. (2016). Neural mechanisms of credit assignment in a multicue environment. J. Neurosci..

[61] Leong Y.C., Radulescu A., Daniel R., DeWoskin V., Niv Y. (2017). Dynamic interaction between reinforcement learning and attention in multidimensional environments. Neuron.

[62] Hallion L.S., Ruscio A.M. (2011). A meta-analysis of the effect of cognitive bias modification on anxiety and depression. Psychol. Bull..

[63] Stolicyn A., Steele J.D., Seriès P. (2016). Conditioned task-set competition: neural mechanisms of emotional interference in depression. Cogn. Affect. Behav. Neurosci..

[64] Rowe G., Hirsh J.B., Anderson A.K. (2007). Positive affect increases the breadth of attentional selection. Proc. Natl. Acad. Sci..

[65] Gable P., Harmon-Jones E. (2010). The motivational dimensional model of affect: implications for breadth of attention, memory, and cognitive categorisation. Cogn. Emot..

[66] Grol M., Koster E.H.W., Bruyneel L., De Raedt R. (2013). Effects of positive mood on attention broadening for self-related information. Psychol. Res..

[67] Eldar E., Cohen J.D., Niv Y. (2013). The effects of neural gain on attention and learning. Nat. Neurosci..

[68] Padoa-Schioppa C., Assad J.A. (2006). Neurons in the orbitofrontal cortex encode economic value. Nature.

[69] Klein-Flugge M.C., Barron H.C., Brodersen K.H., Dolan R.J., Behrens T.E.J. (2013). Segregated encoding of reward-Identity and stimulus-reward associations in human orbitofrontal cortex. J. Neurosci..

[70] Schiffer A.-M., Muller T., Yeung N., Waszak F. (2014). Reward activates stimulus-specific and task-dependent representations in visual association cortices. J. Neurosci..

[71] Boorman E.D., Rajendran V.G., O’Reilly J.X., Behrens T.E. (2016). Two anatomically and computationally distinct learning signals predict changes to stimulus-outcome associations in hippocampus. Neuron.

[72] Noonan M.P., Walton M.E., Behrens T.E.J., Sallet J., Buckley M.J., Rushworth M.F.S. (2010). Separate value comparison and learning mechanisms in macaque medial and lateral orbitofrontal cortex. Proc. Natl. Acad. Sci..

[73] Walton M.E., Behrens T.E.J., Buckley M.J., Rudebeck P.H., Rushworth M.F.S. (2010). Separable learning systems in the macaque brain and the role of orbitofrontal cortex in contingent learning. Neuron.

[74] Walton M.E., Behrens T.E.J., Noonan M.P., Rushworth M.F.S. (2011). Giving credit where credit is due: orbitofrontal cortex and valuation in an uncertain world: orbitofrontal cortex: value assignment and comparison. Ann. N. Y. Acad. Sci..

[75] Noonan M.P., Chau B., Rushworth M.F., Fellows L.K. (2017). Contrasting effects of medial and lateral orbitofrontal cortex lesions on credit assignment and decision making in humans. J. Neurosci..

[76] Noonan M., Mars R., Rushworth M. (2011). Distinct roles of three frontal cortical areas in reward-guided behavior. J. Neurosci..

[77] Boorman E.D., O’Doherty J.P., Adolphs R., Rangel A. (2013). The behavioral and neural mechanisms underlying the tracking of expertise. Neuron.

[78] Chau B.K.H., Sallet J., Papageorgiou G.K., Noonan M.P., Bell A.H., Walton M.E., Rushworth M.F.S. (2015). Contrasting roles for orbitofrontal cortex and amygdala in credit assignment and learning in macaques. Neuron.

[79] Jocham G., Brodersen K.H., Constantinescu A.O., Kahn M.C., Ianni A.M., Walton M.E., Rushworth M.F.S., Behrens T.E.J. (2016). Reward-guided learning with and without causal attribution. Neuron.

[80] Lee S.W., O’Doherty J.P., Shimojo S. (2015). Neural computations mediating one-shot learning in the human brain. PLoS Biol..

[81] Abramson L.Y., Metalsky G.I., Alloy L.B. (1989). Hopelessness depression: a theory-based subtype of depression. Psychol. Rev..

[82] Alloy L.B., Abramson L.Y., Gibb B.E., Crossfield A.G., Pieracci A.M., Spasojevic J., Steinberg J.A. (2004). Developmental antecedents of cognitive vulnerability to depression: review of findings from the cognitive vulnerability to depression project. J. Cogn. Psychother..

[83] Abramson L.Y., Seligman M.E., Teasdale J.D. (1978). Learned helplessness in humans: critique and reformulation. J. Abnorm. Psychol..

[84] Peterson C., Semmel A., Von Baeyer C., Abramson L.Y., Metalsky G.I., Seligman M.E. (1982). The attributional style questionnaire. Cogn. Ther. Res..

[85] Haeffel Gerald J., Gibb Brandon E., Metalsky Gerald I., Alloy Lauren B., Abramson Lyn Y., Hankin Benjamin L., Joiner Thomas E., Swendsen Joel D. (2008). Measuring cognitive vulnerability to depression: Development and validation of the cognitive style questionnaire. Clin. Psychol. Rev..

[86] Hankin B.L., Abramson L.Y., Miller N., Haeffel G.J. (2004). Cognitive vulnerability-stress theories of depression: examining affective specificity in the prediction of depression versus anxiety in three prospective studies. Cogn. Ther. Res..

[87] Haeffel G.J., Gibb B.E., Metalsky G.I., Alloy L.B., Abramson L.Y., Hankin B.L., Joiner T.E., Swendsen J.D. (2008). Measuring cognitive vulnerability to depression: development and validation of the cognitive style questionnaire. Clin. Psychol. Rev..

[88] Alloy L.B., Abramson L.Y. (1979). Judgment of contingency in depressed and nondepressed students: sadder but wiser?. J. Exp. Psychol. Gen..

[89] Msetfi R.M., Murphy R.A., Simpson J. (2007). Depressive realism and the effect of intertrial interval on judgements of zero, positive, and negative contingencies. Q. J. Exp. Psychol..

[90] Msetfi R.M., Wade C., Murphy R.A. (2013). Context and time in causal learning: contingency and mood dependent effects. PLoS One.

[91] Blanco F., Matute H., Vadillo M.A. (2012). Mediating role of activity level in the depressive realism effect. PLoS One.

[92] Byrom N.C., Msetfi R.M., Murphy R.A. (2015). Two pathways to causal control: use and availability of information in the environment in people with and without signs of depression. Acta Psychol (Amst.).

[93] Allan L.G., Siegel S., Hannah S. (2007). The sad truth about depressive realism. Q. J. Exp. Psychol..

[94] Doll B.B., Simon D.A., Daw N.D. (2012). The ubiquity of model-based reinforcement learning. Curr. Opin. Neurobiol..

[95] Huys Q.J.M., Daw N.D., Dayan P. (2015). Depression a decision-theoretic analysis. Annu. Rev. Neurosci..

[96] O’Doherty J.P., Lee S.W., McNamee D. (2015). The structure of reinforcement-learning mechanisms in the human brain. Curr. Opin. Behav. Sci..

[97] Lee S.W., Shimojo S., O’Doherty J.P. (2014). Neural computations underlying arbitration between model-based and model-free learning. Neuron.

[98] Gläscher J., Daw N., Dayan P., O’Doherty J.P. (2010). States versus rewards: dissociable neural prediction error signals underlying model-based and model-free reinforcement learning. Neuron.

[99] Daw N.D., Gershman S.J., Seymour B., Dayan P., Dolan R.J. (2011). Model-based influences on humans’ choices and striatal prediction errors. Neuron.

[100] Lohrenz T., McCabe K., Camerer C.F., Montague P.R. (2007). Neural signature of fictive learning signals in a sequential investment task. Proc. Natl. Acad. Sci..

[101] Boorman E.D., Behrens T.E.J., Woolrich M.W., Rushworth M.F.S. (2009). How green is the grass on the other side? Frontopolar cortex and the evidence in favor of alternative courses of action. Neuron.

[102] Scholl J., Kolling N., Nelissen N., Wittmann M.K., Harmer C.J., Rushworth M.F. (2015). The good, the bad, and the irrelevant: neural mechanisms of learning real and hypothetical rewards and effort. J. Neurosci..

[103] Vickery T.J., Chun M.M., Lee D. (2011). Ubiquity and specificity of reinforcement signals throughout the human brain. Neuron.

[104] Volman I., Roelofs K., Koch S., Verhagen L., Toni I. (2011). Anterior prefrontal cortex inhibition impairs control over social emotional actions. Curr. Biol..

[105] Volman I., Verhagen L., den Ouden H.E., Fernández G., Rijpkema M., Franke B., Toni I., Roelofs K. (2013). Reduced serotonin transporter availability decreases prefrontal control of the amygdala. J. Neurosci..

[106] Volman I., Katinka Louise von Borries A., Hendrik Bulten B., Jan Verkes R., Toni I., Roelofs K. (2016). Testosterone modulates altered prefrontal control of emotional actions in psychopathic offenders. eNeuro.

[107] Vaidya A.R., Fellows L.K. (2016). Necessary contributions of human frontal lobe subregions to reward learning in a dynamic, multidimensional environment. J. Neurosci..

[108] Beck A.T. (2008). The evolution of the cognitive model of depression and its neurobiological correlates. Am. J. Psychiatry.

[109] Clark D.A., Beck A.T. (2010). Cognitive theory and therapy of anxiety and depression: convergence with neurobiological findings. Trends Cogn. Sci..

[110] Disner S.G., Beevers C.G., Haigh E.A.P., Beck A.T. (2011). Neural mechanisms of the cognitive model of depression. Nat. Rev. Neurosci..

[111] Gershman S.J., Niv Y. (2010). Learning latent structure: carving nature at its joints. Curr. Opin. Neurobiol..

[112] Wilson R.C., Takahashi Y.K., Schoenbaum G., Niv Y. (2014). Orbitofrontal cortex as a cognitive map of task space. Neuron.

[113] Chan S.C.Y., Niv Y., Norman K.A. (2016). A probability distribution over latent causes, in the orbitofrontal cortex. J. Neurosci..

[114] Constantinescu A.O., O’Reilly J.X., Behrens T.E. (2016). Organizing conceptual knowledge in humans with a gridlike code. Science.

[115] Schuck N.W., Cai M.B., Wilson R.C., Niv Y. (2016). Human orbitofrontal cortex represents a cognitive map of state space. Neuron.

[116] Amiez C. (2005). Reward encoding in the monkey anterior cingulate cortex. Cereb. Cortex.

[117] Kennerley S.W., Walton M.E., Behrens T.E.J., Buckley M.J., Rushworth M.F.S. (2006). Optimal decision making and the anterior cingulate cortex. Nat. Neurosci..

[118] Karlsson M.P., Tervo D.G., Karpova A.Y. (2012). Network resets in medial prefrontal cortex mark the onset of behavioral uncertainty. Science.

[119] Kolling N., Behrens T.E., Mars R.B., Rushworth M.F. (2012). Neural mechanisms of foraging. Science.

[120] Kolling N., Wittmann M., Rushworth M.F.S. (2014). Multiple neural mechanisms of decision making and their competition under changing risk pressure. Neuron.

[121] O’Reilly J.X., Schüffelgen U., Cuell S.F., Behrens T.E., Mars R.B., Rushworth M.F. (2013). Dissociable effects of surprise and model update in parietal and anterior cingulate cortex. Proc. Natl. Acad. Sci..

[122] Tervo D.G.R., Proskurin M., Manakov M., Kabra M., Vollmer A., Branson K., Karpova A.Y. (2014). Behavioral variability through stochastic choice and its gating by anterior cingulate cortex. Cell.

[123] Doll B.B., Duncan K.D., Simon D.A., Shohamy D., Daw N.D. (2015). Model-based choices involve prospective neural activity. Nat. Neurosci..

[124] Schuck N.W., Gaschler R., Wenke D., Heinzle J., Frensch P.A., Haynes J.-D., Reverberi C. (2015). Medial prefrontal cortex predicts internally driven strategy shifts. Neuron.

[125] Wittmann M.K., Kolling N., Akaishi R., Chau B.K., Brown J.W., Nelissen N., Rushworth M.F. (2016). Predictive decision making driven by multiple time-linked reward representations in the anterior cingulate cortex. Nat. Commun..

[126] Scholl J., Kolling N., Nelissen N., Stagg C.J., Harmer C.J., Rushworth M.F. (2017). Excitation and inhibition in anterior cingulate predict use of past experiences. eLife.

[127] Stephan K.E., Manjaly Z.M., Mathys C.D., Weber L.A.E., Paliwal S., Gard T., Tittgemeyer M., Fleming S.M., Haker H., Seth A.K., Petzschner F.H. (2016). Allostatic self-efficacy.. a metacognitive theory of dyshomeostasis-induced fatigue and depression. Front. Hum. Neurosci..

[128] Drysdale A.T., Grosenick L., Downar J., Dunlop K., Mansouri F., Meng Y., Fetcho R.N., Zebley B., Oathes D.J., Etkin A., Schatzberg A.F., Sudheimer K., Keller J., Mayberg H.S., Gunning F.M., Alexopoulos G.S., Fox M.D., Pascual-Leone A., Voss H.U., Casey B.J., Dubin M.J., Liston C. (2017). Resting-state connectivity biomarkers define neurophysiological subtypes of depression. Nat. Med..

[129] Rushworth M.F.S., Noonan M.P., Boorman E.D., Walton M.E., Behrens T.E. (2011). Frontal cortex and reward-guided learning and decision-making. Neuron.

[130] Rushworth M.F., Kolling N., Sallet J., Mars R.B. (2012). Valuation and decision-making in frontal cortex: one or many serial or parallel systems?. Curr Opin. Neurobiol..

[131] Öngür D., Price J.L. (2000). The organization of networks within the orbital and medial prefrontal cortex of rats, monkeys and humans. Cereb. Cortex.

[132] Schoenbaum G., Chiba A.A., Gallagher M. (1998). Orbitofrontal cortex and basolateral amygdala encode expected outcomes during learning. Nat. Neurosci..

[133] Schoenbaum G., Chiba A.A., Gallagher M. (1999). Neural encoding in orbitofrontal cortex and basolateral amygdala during olfactory discrimination learning. J. Neurosci..

[134] Tremblay L., Schultz W. (1999). Relative reward preference in primate orbitofrontal cortex. Nature.

[135] Hikosaka K., Watanabe M. (1991). Delay activity of orbital and lateral prefrontal neurons of the monkey varying with different rewards. Cereb. Cortex N. Y. N.

[136] Wallis J.D., Miller E.K. (2003). Neuronal activity in primate dorsolateral and orbital prefrontal cortex during performance of a reward preference task. Eur. J. Neurosci..

[137] Roesch M.R., Olson C.R. (2004). Neuronal activity related to reward value and motivation in primate frontal cortex. Science.

[138] Rudebeck P.H., Behrens T.E., Kennerley S.W., Baxter M.G., Buckley M.J., Walton M.E., Rushworth M.F.S. (2008). Frontal cortex subregions play distinct roles in choices between actions and stimuli. J. Neurosci..

[139] Kennerley S.W., Dahmubed A.F., Lara A.H., Wallis J.D. (2009). Neurons in the frontal lobe encode the value of multiple decision variables. J. Cogn. Neurosci..

[140] Wang M.Z., Hayden B.Y. (2017). Reactivation of associative structure specific outcome responses during prospective evaluation in reward-based choices. Nat. Commun..

[141] Hayden B.Y., Platt M.L. (2010). Neurons in anterior cingulate cortex multiplex information about reward and action. J. Neurosci..

[142] Lau B., Glimcher P.W. (2008). Value representations in the primate striatum during matching behavior. Neuron.

[143] Matsumoto K., Suzuki W., Tanaka K. (2003). Neuronal correlates of goal-based motor selection in the prefrontal cortex. Science.

[144] Samejima K., Ueda Y., Doya K., Kimura M. (2005). Representation of action-specific reward values in the striatum. Science.

[145] Camille N., Tsuchida A., Fellows L.K. (2011). Double dissociation of stimulus-value and action-value learning in humans with orbitofrontal or anterior cingulate cortex damage. J. Neurosci..

[146] Dum R.P., Strick P.L. (1996). Spinal cord terminations of the medial wall motor areas in macaque monkeys. J. Neurosci..

[147] Morecraft R.J., Van Hoesen G.W. (1993). Frontal granular cortex input to the cingulate (M3), supplementary (M2) and primary (M1) motor cortices in the rhesus monkey. J. Comp. Neurol..

[148] Hunt L.T., Dolan R.J., Behrens T.E.J. (2014). Hierarchical competitions subserving multi-attribute choice. Nat. Neurosci..

[149] Meyer-Lindenberg A., Tost H. (2012). Neural mechanisms of social risk for psychiatric disorders. Nat. Neurosci..

[150] Rushworth M.F., Mars R.B., Sallet J. (2013). Are there specialized circuits for social cognition and are they unique to humans?. Curr Opin. Neurobiol..

[151] Ruff C.C., Fehr E. (2014). The neurobiology of rewards and values in social decision making. Nat. Rev. Neurosci..

[152] Eshel N., Roiser J.P. (2010). Reward and punishment processing in depression. Biol. Psychiatry.

[153] Roiser J.P., Sahakian B.J. (2013). Hot and cold cognition in depression. CNS Spectr..

[154] Amsterdam J.D., Settle R.G., Doty R.L., Abelman E., Winokur A. (1987). Taste and smell perception in depression. Biol. Psychiatry.

[155] Berlin I., Givry-Steiner L., Lecrubier Y., Puech A.J. (1998). Measures of anhedonia and hedonic responses to sucrose in depressive and schizophrenic patients in comparison with healthy subjects. Eur. Psychiatry J. Assoc. Eur. Psychiatr..

[156] Sherdell L., Waugh C.E., Gotlib I.H. (2012). Anticipatory pleasure predicts motivation for reward in major depression. J. Abnorm. Psychol..

[157] Chung D., Kadlec K., Aimone J.A., McCurry K., King-Casas B., Chiu P.H. (2017). Valuation in major depression is intact and stable in a non-learning environment. Sci. Rep..

[158] Rottenberg J., Kasch K.L., Gross J.J., Gotlib I.H. (2002). Sadness and amusement reactivity differentially predict concurrent and prospective functioning in major depressive disorder. Emot. Wash. DC.

[159] Beats B.C., Sahakian B.J., Levy R. (1996). Cognitive performance in tests sensitive to frontal lobe dysfunction in the elderly depressed. Psychol. Med..

[160] Elliott R., Sahakian B.J., McKay A.P., Herrod J.J., Robbins T.W., Paykel E.S. (1996). Neuropsychological impairments in unipolar depression: the influence of perceived failure on subsequent performance. Psychol. Med..

[161] Steele J.D., Kumar P., Ebmeier K.P. (2007). Blunted response to feedback information in depressive illness. Brain.

[162] Bylsma L.M., Morris B.H., Rottenberg J. (2008). A meta-analysis of emotional reactivity in major depressive disorder. Clin. Psychol. Rev..

[163] Dolan R.J., Dayan P. (2013). Goals and habits in the brain. Neuron.

[164] Guitart-Masip M., Duzel E., Dolan R., Dayan P. (2014). Action versus valence in decision making. Trends Cogn. Sci..

[165] Guitart-Masip M., Huys Q.J.M., Fuentemilla L., Dayan P., Duzel E., Dolan R.J. (2012). Go and no-go learning in reward and punishment: interactions between affect and effect. NeuroImage.

[166] Amemori K., Graybiel A.M. (2012). Localized microstimulation of primate pregenual cingulate cortex induces negative decision-making. Nat. Neurosci..

[167] Aggleton J.P., Wright N.F., Rosene D.L., Saunders R.C. (1991). Complementary patterns of direct amygdala and hippocampal projections to the macaque prefrontal cortex. Cereb. Cortex N. Y. N.

[168] Price J.L., Drevets W.C. (2010). Neurocircuitry of mood disorders. Neuropsychopharmacology.

[169] Huys Q.J.M., Cools R., Gölzer M., Friedel E., Heinz A., Dolan R.J., Dayan P. (2011). Disentangling the roles of approach, activation and valence in instrumental and pavlovian responding. PLoS Comput. Biol..

[170] Holland P.C., Gallagher M. (2003). Double dissociation of the effects of lesions of basolateral and central amygdala on conditioned stimulus-potentiated feeding and Pavlovian-instrumental transfer. Eur. J. Neurosci..

[171] Corbit L.H., Balleine B.W. (2005). Double dissociation of basolateral and central amygdala lesions on the general and outcome-specific forms of pavlovian-instrumental transfer. J. Neurosci..

[172] Talmi D., Seymour B., Dayan P., Dolan R.J. (2008). Human pavlovian-instrumental transfer. J. Neurosci..

[173] Prévost C., Liljeholm M., Tyszka J.M., O’Doherty J.P. (2012). Neural correlates of specific and general pavlovian-to-instrumental transfer within human amygdalar subregions: a high-resolution fMRI study. J. Neurosci..

[174] Lebreton M., Jorge S., Michel V., Thirion B., Pessiglione M. (2009). An automatic valuation system in the human brain: evidence from functional neuroimaging. Neuron.

[175] Huys Q.J.M., Gölzer M., Friedel E., Heinz A., Cools R., Dayan P., Dolan R.J. (2016). The specificity of Pavlovian regulation is associated with recovery from depression. Psychol. Med..

[176] Bach D.R., Guitart-Masip M., Packard P.A., Miró J., Falip M., Fuentemilla L., Dolan R.J. (2014). Human hippocampus arbitrates approach-avoidance conflict. Curr. Biol. CB.

[177] Geurts D.E.M., Huys Q.J.M., den Ouden H.E.M., Cools R. (2013). Serotonin and aversive Pavlovian control of instrumental behavior in humans. J. Neurosci..

[178] Drevets W.C., Price J.L., Simpson J.R., Todd R.D., Reich T., Vannier M., Raichle M.E. (1997). Subgenual prefrontal cortex abnormalities in mood disorders. Nature.

[179] Botteron K.N., Raichle M.E., Drevets W.C., Heath A.C., Todd R.D. (2002). Volumetric reduction in left subgenual prefrontal cortex in early onset depression. Biol. Psychiatry.

[180] Drevets W.C. (2003). Neuroimaging abnormalities in the amygdala in mood disorders. Ann. N. Y. Acad. Sci..

[181] Admon R., Kaiser R.H., Dillon D.G., Beltzer M., Goer F., Olson D.P., Vitaliano G., Pizzagalli D.A. (2016). Dopaminergic enhancement of striatal response to reward in major depression. Am. J. Psychiatry..

[182] Eldar E., Niv Y. (2015). Interaction between emotional state and learning underlies mood instability. Nat. Commun..

[183] Eldar E., Rutledge R.B., Dolan R.J., Niv Y. (2016). Mood as representation of momentum. Trends Cogn. Sci..

[184] Kurniawan I.T., Seymour B., Talmi D., Yoshida W., Chater N., Dolan R.J. (2010). Choosing to make an effort: the role of striatum in signaling physical effort of a chosen action. J. Neurophysiol..

[185] Klein-Flügge M.C., Kennerley S.W., Friston K., Bestmann S. (2016). Neural signatures of value comparison in human cingulate cortex during decisions requiring an effort-reward trade-off. J. Neurosci..

[186] Lockwood P.L., Hamonet M., Zhang S.H., Ratnavel A., Salmony F.U., Husain M., Apps M.A.J. (2017). Prosocial apathy for helping others when effort is required. Nat. Hum. Behav..

[187] Hunt L.T., Kolling N., Soltani A., Woolrich M.W., Rushworth M.F.S., Behrens T.E.J. (2012). Mechanisms underlying cortical activity during value-guided choice. Nat. Neurosci..

[188] Kable J.W., Glimcher P.W. (2007). The neural correlates of subjective value during intertemporal choice. Nat. Neurosci..

[189] Nicolle A., Klein-Flügge M.C., Hunt L.T., Vlaev I., Dolan R.J., Behrens T.E.J. (2012). An agent independent axis for executed and modeled choice in medial prefrontal cortex. Neuron.

[190] Prévost C., Pessiglione M., Météreau E., Cléry-Melin M.-L., Dreher J.-C. (2010). Separate valuation subsystems for delay and effort decision costs. J. Neurosci..

[191] Walton M.E., Bannerman D.M., Alterescu K., Rushworth M.F.S. (2003). Functional specialization within medial frontal cortex of the anterior cingulate for evaluating effort-related decisions. J. Neurosci..

[192] Walton M.E., Kennerley S.W., Bannerman D.M., Phillips P.E.M., Rushworth M.F.S. (2006). Weighing up the benefits of work: behavioral and neural analyses of effort-related decision making. Neural Netw..

[193] Rudebeck P.H., Walton M.E., Smyth A.N., Bannerman D.M., Rushworth M.F.S. (2006). Separate neural pathways process different decision costs. Nat. Neurosci..

[194] Croxson P.L., Walton M.E., O’Reilly J.X., Behrens T.E.J., Rushworth M.F.S. (2009). Effort-based cost-benefit valuation and the human brain. J. Neurosci..

[195] Hillman K.L., Bilkey D.K. (2010). Neurons in the rat anterior cingulate cortex dynamically encode cost-benefit in a spatial decision-making task. J. Neurosci..

[196] Hosokawa T., Kennerley S.W., Sloan J., Wallis J.D. (2013). Single-neuron mechanisms underlying cost-benefit analysis in frontal cortex. J. Neurosci..

[197] Bonnelle V., Manohar S., Behrens T., Husain M. (2016). Individual differences in premotor brain systems underlie behavioral apathy. Cereb. Cortex N. Y. NY..

[198] Treadway M.T., Bossaller N.A., Shelton R.C., Zald D.H. (2012). Effort-based decision-making in major depressive disorder: a translational model of motivational anhedonia. J. Abnorm. Psychol..

[199] Cléry-Melin M.-L., Schmidt L., Lafargue G., Baup N., Fossati P., Pessiglione M. (2011). Why don’t you try harder? An investigation of effort production in major depression. PLoS One.

[200] Hartmann M.N., Hager O.M., Reimann A.V., Chumbley J.R., Kirschner M., Seifritz E., Tobler P.N., Kaiser S. (2014). Apathy but not diminished expression in schizophrenia is associated with discounting of monetary rewards by physical effort. Schizophr. Bull..

[201] Bonnelle V., Veromann K.-R., Burnett Heyes S., Lo Sterzo E., Manohar S., Husain M. (2015). Characterization of reward and effort mechanisms in apathy. J. Physiol. Paris.

[202] Fried E.I., Epskamp S., Nesse R.M., Tuerlinckx F., Borsboom D. (2016). What are good depression symptoms? Comparing the centrality of DSM and non-DSM symptoms of depression in a network analysis. J. Affect. Disord..

[203] Saaltink D.-J., Vreugdenhil E. (2014). Stress, glucocorticoid receptors, and adult neurogenesis: a balance between excitation and inhibition?. Cell Mol. Life Sci. CMLS.

[204] Maya Vetencourt J.F., Sale A., Viegi A., Baroncelli L., De Pasquale R., O’Leary O.F., Castrén E., Maffei L. (2008). The antidepressant fluoxetine restores plasticity in the adult visual cortex. Science.

[205] Hasler G., van der Veen J.W., Tumonis T., Meyers N., Shen J., Drevets W.C. (2007). Reduced prefrontal glutamate/glutamine and gamma-aminobutyric acid levels in major depression determined using proton magnetic resonance spectroscopy. Arch. Gen. Psychiatry.

[206] Gabbay V., Mao X., Klein R.G., Ely B.A., Babb J.S., Panzer A.M., Alonso C.M., Shungu D.C. (2012). Anterior cingulate cortex γ-aminobutyric acid in depressed adolescents: relationship to anhedonia. Arch. Gen. Psychiatry.

[207] Yüksel C., Öngür D. (2010). Magnetic resonance spectroscopy studies of glutamate-related abnormalities in mood disorders. Biol. Psychiatry.

[208] Luykx J.J., Laban K.G., van den Heuvel M.P., Boks M.P.M., Mandl R.C.W., Kahn R.S., Bakker S.C. (2012). Region and state specific glutamate downregulation in major depressive disorder: a meta-analysis of (1)H-MRS findings. Neurosci. Biobehav. Rev..

[209] Scholl J., Günthner J., Kolling N., Favaron E., Rushworth M.F., Harmer C.J., Reinecke A. (2014). A role beyond learning for NMDA receptors in reward-based decision-making-a pharmacological study using d-cycloserine. Neuropsychopharmacology.

[210] Binns K.E., Salt T.E. (1996). Importance of NMDA receptors for multimodal integration in the deep layers of the cat superior colliculus. J. Neurophysiol..

[211] American Psychiatric Association (2000). Diagnostic and Statistical Manual of Mental Disorders.

[212] Radford M.H., Mann L., Kalucy R.S. (1986). Psychiatric disturbance and decision-making. Aust. N. Z. J. Psychiatry.

[213] Radford M.H., Nakane Y., Ohta Y., Mann L., Kalucy R.S. (1991). Decision making in clinically depressed patients. A transcultural social psychological study. J. Nerv. Ment. Dis..

[214] Saunders D.E., Peterson G.W., Sampson J.P., Reardon R.C. (2000). Relation of depression and dysfunctional career thinking to career indecision. J. Vocat. Behav..

[215] Okwumabua J.O., Wong S.P., Duryea E.J. (2003). Depressive symptoms and decision making among African American youth. J. Adolesc. Res..

[216] Leykin Y., Roberts C.S., Derubeis R.J. (2011). Decision-making and depressive symptomatology. Cogn. Ther. Res..

[217] Jocham G., Hunt L.T., Near J., Behrens T.E.J. (2012). A mechanism for value-guided choice based on the excitation-inhibition balance in prefrontal cortex. Nat. Neurosci..

[218] Hämmerer D., Bonaiuto J., Klein-Flügge M., Bikson M., Bestmann S. (2016). Selective alteration of human value decisions with medial frontal tDCS is predicted by changes in attractor dynamics. Sci. Rep..

[219] Wang X.-J. (2002). Probabilistic decision making by slow reverberation in cortical circuits. Neuron.

[220] Wang X.-J. (2008). Decision making in recurrent neuronal circuits. Neuron.

[221] Murphy F.C., Rubinsztein J.S., Michael A., Rogers R.D., Robbins T.W., Paykel E.S., Sahakian B.J. (2001). Decision-making cognition in mania and depression. Psychol. Med..

[222] Green C.S., Benson C., Kersten D., Schrater P. (2010). Alterations in choice behavior by manipulations of world model. Proc. Natl. Acad. Sci. U. S. A..

[223] Daw N.D., O’Doherty J.P., Dayan P., Seymour B., Dolan R.J. (2006). Cortical substrates for exploratory decisions in humans. Nature.

[224] Wilson R.C., Geana A., White J.M., Ludvig E.A., Cohen J.D. (2014). Humans use directed and random exploration to solve the explore-exploit dilemma. J. Exp. Psychol. Gen..

[225] Zajkowski W.K., Kossut M., Wilson R.C. (2017). A causal role for right frontopolar cortex in directed, but not random, exploration. bioRxiv.

[226] Boorman E.D., Rushworth M.F., Behrens T.E. (2013). Ventromedial prefrontal and anterior cingulate cortex adopt choice and default reference frames during sequential multi-alternative choice. J. Neurosci..

[227] Quilodran R., Rothé M., Procyk E. (2008). Behavioral shifts and action valuation in the anterior cingulate cortex. Neuron.

[228] Hayden B.Y., Pearson J.M., Platt M.L. (2011). Neuronal basis of sequential foraging decisions in a patchy environment. Nat. Neurosci..

[229] Seo H., Cai X., Donahue C.H., Lee D. (2014). Neural correlates of strategic reasoning during competitive games. Science.

[230] Blanco N.J., Otto A.R., Maddox W.T., Beevers C.G., Love B.C. (2013). The influence of depression symptoms on exploratory decision-making. Cognition.

[231] Gläscher J.P., O’Doherty J.P. (2010). Model-based approaches to neuroimaging: combining reinforcement learning theory with fMRI data. Wiley Interdiscip. Rev. Cogn. Sci..

[232] Schwartenbeck P., Friston K. (2016). Computational phenotyping in psychiatry: a worked example. Eneuro.

[233] Daw N.D. (2011). Trial-by-trial data analysis using computational models. Decis. Mak. Affect. Learn. Atten. Perform..

[234] Nassar M.R., Gold J.I. (2013). A healthy fear of the unknown: perspectives on the interpretation of parameter fits from computational models in neuroscience. PLoS Comput. Biol..

[235] Palminteri S., Wyart V., Koechlin E. (2017). The importance of falsification in computational cognitive modeling. Trends Cogn. Sci..

[236] Vogt B. (2009). Cingulate Neurobiology and Disease.

[237] Procyk E., Wilson C.R.E., Stoll F.M., Faraut M.C.M., Petrides M., Amiez C. (1991). Midcingulate motor map and feedback detection: converging data from humans and monkeys. Cereb. Cortex N. Y. N.

[238] Vogt B.A., Nimchinsky E.A., Vogt L.J., Hof P.R. (1995). Human cingulate cortex: surface features, flat maps, and cytoarchitecture. J. Comp. Neurol..

[239] Sallet J., Mars R.B., Noonan M.P., Neubert F.-X., Jbabdi S., O’Reilly J.X., Filippini N., Thomas A.G., Rushworth M.F. (2013). The organization of dorsal frontal cortex in humans and macaques. J. Neurosci..

[240] Neubert F.-X., Mars R.B., Thomas A.G., Sallet J., Rushworth M.F.S. (2014). Comparison of human ventral frontal cortex areas for cognitive control and language with areas in monkey frontal cortex. Neuron.

[241] Neubert F.-X., Mars R.B., Sallet J., Rushworth M.F.S. (2015). Connectivity reveals relationship of brain areas for reward-guided learning and decision making in human and monkey frontal cortex. Proc. Natl. Acad. Sci. U. S. A..

[242] Barron H.C., Garvert M.M., Behrens T.E.J. (2016). Repetition suppression: a means to index neural representations using BOLD?. Philos. Trans. R. Soc. B.

[243] Tversky A., Kahneman D. (1992). Advances in prospect theory: cumulative representation of uncertainty. J. Risk Uncertain.

[244] Kahneman D., Tversky A. (1979). Prospect theory − analysis of decision under risk. Econometrica.

[245] Prelec D. (1998). The probability weighting function. Econometrica.

[246] Hartmann M.N., Hager O.M., Tobler P.N., Kaiser S. (2013). Parabolic discounting of monetary rewards by physical effort. Behav. Processes.

[247] Klein-Flügge M.C., Kennerley S.W., Saraiva A.C., Penny W.D., Bestmann S. (2015). Behavioral modeling of human choices reveals dissociable effects of physical effort and temporal delay on reward devaluation. PLoS Comput. Biol..

[248] Myerson J., Green L. (1995). Discounting of delayed rewards: models of individual choice. J. Exp. Anal. Behav..

[249] Madden G.J., Bickel W.K., Jacobs E.A. (1999). Discounting of delayed rewards in opioid-dependent outpatients: exponential or hyperbolic discounting functions?. Exp. Clin. Psychopharmacol..

[250] Stagg C.J., Nitsche M.A. (2011). Physiological basis of transcranial direct current stimulation. Neuroscientist.

[251] Rahman A., Reato D., Arlotti M., Gasca F., Datta A., Parra L.C., Bikson M. (2013). Cellular effects of acute direct current stimulation: somatic and synaptic terminal effects. J. Physiol..

[252] Sharot Tali, Christoph W. Korn, Raymond J. Dolan (2011). How unrealistic optimism is maintained in the face of reality. Nature neuroscience.

